# Biomimetic nanoparticles drive the mechanism understanding of shear-wave elasticity stiffness in triple negative breast cancers to predict clinical treatment

**DOI:** 10.1016/j.bioactmat.2022.10.025

**Published:** 2022-11-03

**Authors:** Dongdong Zheng, Jin Zhou, Lang Qian, XueJiao Liu, Cai Chang, Shuang Tang, Hongbo Zhang, Shichong Zhou

**Affiliations:** a Department of Ultrasound, Fudan University Shanghai Cancer Center, Shanghai, 200032, PR China; b Pharmaceutical Sciences Laboratory, Åbo Akademi University, FI-00520, Turku, Finland; c Cancer Institute, Fudan University Shanghai Cancer Center, Shanghai, 200032, PR China; d Institutes of Biomedical Sciences and Department of Chemistry, Fudan University, Shanghai, 200032, PR China; e Department of Nuclear Medicine, Fudan University Shanghai Cancer Center, Shanghai, 200032, PR China; f Turku Bioscience Center, University of Turku and Åbo Akademi University, FI-00520, Turku, Finland; g The First Affiliated Hospital of Wenzhou Medical University, Wenzhou, 325000, PR China

**Keywords:** Cancer-associated fibroblasts, Biomimetic nanoparticles, Shear-wave elasticity imaging, Theranostic prediction

## Abstract

In clinical practice, we noticed that triple negative breast cancer (TNBC) patients had higher shear-wave elasticity (SWE) stiffness than non-TNBC patients and a higher α-SMA expression was found in TNBC tissues than the non-TNBC tissues. Moreover, SWE stiffness also shows a clear correlation to neoadjuvant response efficiency. To elaborate this phenomenon, TNBC cell membrane-modified polylactide acid-glycolic acid (PLGA) nanoparticle was fabricated to specifically deliver artesunate to regulate SWE stiffness through inhibiting CAFs functional status. As tested in MDA-MB-231 and E0771 orthotopic tumor models, CAFs functional status inhibited by 231M-ARS@PLGA nanoparticles (231M-AP NPs) had reduced the SWE stiffness as well as attenuated hypoxia of tumor as tumor soil loosening agent which amplified the antitumor effects of paclitaxel and PD1 inhibitor. Single-cell sequencing indicated that the two main CAFs (extracellular matrix and wound healing CAFs) that produces extracellular matrix could influence the tumor SWE stiffness as well as the antitumor effect of drugs. Further, biomimetic nanoparticles inhibited CAFs function could attenuate tumor hypoxia by increasing proportion of inflammatory blood vessels and oxygen transport capacity. Therefore, our finding is fundamental for understanding the role of CAFs on affecting SWE stiffness and drugs antitumor effects, which can be further implied in the potential clinical theranostic predicting in neoadjuvant therapy efficacy through non-invasive analyzing of SWE imaging.

## Highlights

• Tumor nano-soil loosening agent could regulate SWE imaging stiffness of TNBC tumor by keeping CAFs in a resting state.
• The CAFs functional status affect the antitumor effect of chemotherapy and immunotherapy for TNBC.
• Two subtypes of CAFs (Extracellular matrix CAFs and wound healing CAFs) mainly affect the SWE imaging stiffness of TNBC.
• SWE imaging stiffness could predict the response of patients to neoadjuvant therapy in clinical.
• Tumor nano-soil loosening agent has good biosafety and targeting effect.

## Graphical abstract

**Figure:**
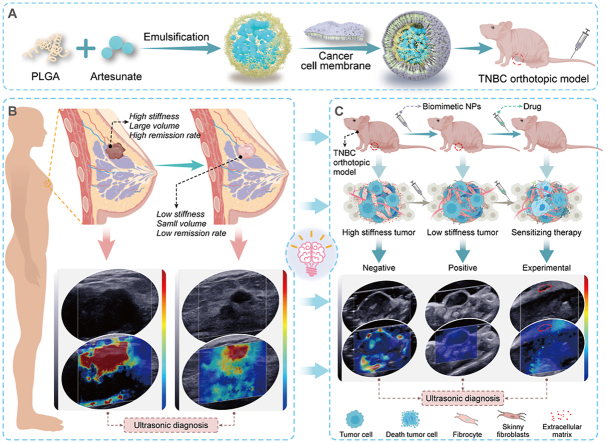

## 1 Introduction

Cancer-associated fibroblasts (CAFs), as a component in solid tumors, play an important role in tumor development and metastasis [1]. CAFS, together with their secreted collagen constitute a major part of solid tumors [2,3]. The tumor microenvironment (TME), comprising CAFs and tumor-associated macrophages (TAM), is conducive for the survival and progress of cancer cells [4,5]. When the tumor undergoes traumatic stress, CAFs in the solid tumor are activated and they release proteolytic enzymes, cytokines, and chemokines; these facilitate tumor invasion, drug resistance, and immunosuppression [6,7]. Several cancers (e.g., pancreatic cancer, breast cancer, and liver cancer) exhibit a high extent of fibrosis in the tumor [8,9]. Therefore, CAFs could be an important component of solid tumors; however, these have been ignored in the context of chemotherapy and immune therapy.

Breast cancer, a kind of the most common malignant tumors in women worldwide, is divided into three pathological types [10]. Triple negative breast cancer (TNBC), a type of breast cancer with negative expression of estrogen receptor (ER), progesterone receptor (PR), and human epidermal growth factor receptor 2 (HER2), is characterized by rapid progress, metastasis, and invasion tendency. Most patients are already in the advanced stage when they are clinically diagnosed as TNBC. Some of these patients require neoadjuvant therapy before surgery. However, the response rate to neoadjuvant therapy is limited, and in certain cases, the tumor volume continues to increase during the treatment. Therefore, it is important to develop a clinical tool to predict the sensitivity of patients to neoadjuvant therapy, and it can help clinicians to formulate treatment plans [11,12]. Generally, tumor histopathological indicators are utilized as reference for this purpose. However, a minimally invasive or non-invasive pre-examination is preferred. Ultrasound-based methods are valuable for breast cancer diagnosis, among which shear-wave elastography (SWE) imaging has wide applications. The SWE imaging features have a correlation with the tumor pathological features. The SWE stiffness of breast cancer is related to the degree of tumor hypoxia [13]. In addition, the SWE stiffness of TNBC tumors is higher than that of non-TNBC tumors [14]. TNBC tumors have a worse TME than non-TNBC tumors (e.g., lower percentage CD8^+^ cells infiltration), this contributes to the high metastasis and invasion in TNBC [15]. A terrible TME is often associated with tumor drug resistance and immunosuppression [2,16,17]. Therefore, the non-invasive SWE imaging has a clinical significance to diagnosis the pathological indicators of TNBC and predict the sensitivity of TNBC to chemotherapy or immunotherapy, and to assist clinicians in developing personalized treatment plans [18].

SWE imaging is a process in which different shear wave are generated when ultrasonic wave tapped on different elastic coefficient tissues. CAFs account for the majority of solid tumors [19], SWE imaging is naturally correlated to CAFs. Some researchers [20,21] believed that in liver, SWE stiffness is closely related to the degree of liver fibrosis in cirrhosis. In addition, it has been reported that SWE stiffness is associated with CAFs content in breast cancer [22]. Based on physiological cognition and clinical observation, we find that both inflammation (in a sense, tumor is a chronic inflammatory response) and tumor exhibit more intensive fibrosis during the repair or at the final cure stage [23]. The extent of intratumoral fibrosis increases as the treatment progresses. However, in clinical practice, SWE stiffness decreases with the treatment progress, which is contradictory to the above hypothesis. Current explanations do not fully explain this phenomenon clearly. This phenomenon is clinically significant, but the underlying mechanism needs to be elucidated.

Artesunate (ARS), a derivative of artemisinin, is promising for its anti-tumor potential. ARS is a STAT3 inhibitor and inhibits the JAK-STAT3 pathway and reduces STAT3 phosphorylation, inducing apoptosis of tumor cells [24]. JAK-STAT3 pathway was also associated with TGF-β1 pathway [25]. ARS sensitizes tumors to chemotherapy [26], which could be related to the anti-fibrosis effect of ARS [25]. Combining ARS with chemotherapeutic or immunotherapeutic agents interferes with CAFs and cancer cells simultaneously [27], may enable better treatment outcomes. However, pharmacokinetic analyses of artesunate after intravenous administration in humans indicated that it has a short half-life [28]. This limits its potential as an antitumor agent.

In recent years, nanomedicine has developed rapidly [29,30], and biomimetic nanoparticles with tumor cell membranes have been widely used to improve the circulation and tumor targeting ability of nanomedicine *in vivo* [31,32]. For instance, galectin-1, galectin-3, and CD47 are expressed on the tumor cell membrane. Galectin-1 and galectin-3 make tumor cells prone to homologous aggregation [33,34], while CD47 allows tumor cells to escape phagocytosis by immune cells [35,36], thus improving tumor cell circulation *in vivo*. Therefore, engineering biomimetic nanoparticles to enhance the anti-fibrosis effect of ARS on tumor and to remodel TME could help us to better understand the relationship between SWE stiffness and CAFs.

**Scheme. 1:**
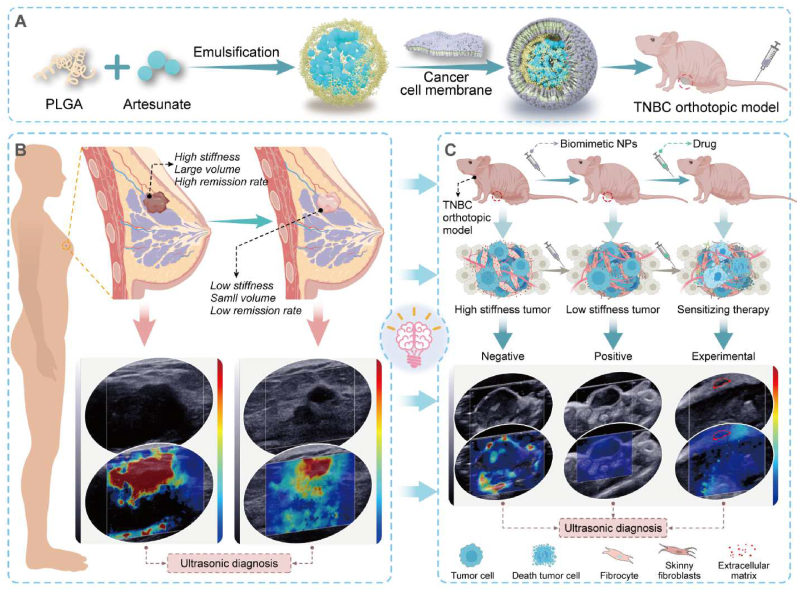
Schematic illustration of this study outline. (A) The preparation procedure of biomimetic nanoparticles. **(B&C)** Based on clinical data analysis, we established MDA-MB-231 and E0771 orthotopic tumor models to explore the mechanism by which shear wave elastic imaging can predict TNBC treatment efficacy.

In this work, we analyzed the SWE images and pathological indicators of 233 patients, and the breast cancer tissue of 100 patients. We established a preliminary SWE prediction model based on the data from 14 TNBC patients, who received neoadjuvant therapy. Further, we prepared engineered biomimetic nanoparticles and applied it to MDA-MB-231 orthotopic xenograft tumor model and E0771 orthotopic tumor model to elucidate the mechanism of determining tumor SWE imaging stiffness. We also used single cell sequencing to further elucidate the mechanism and validated the results *in vivo*. In conclusion, we have provided a comprehensive description of the mechanism of breast cancer SWE imaging stiffness and a SWE imaging-based prediction model for neoadjuvant therapy efficiency (Scheme 1).

## 2 Results

### 2.1 SWE imaging predicted neoadjuvant response efficiency in TNBC patients

**Fig. 1:**
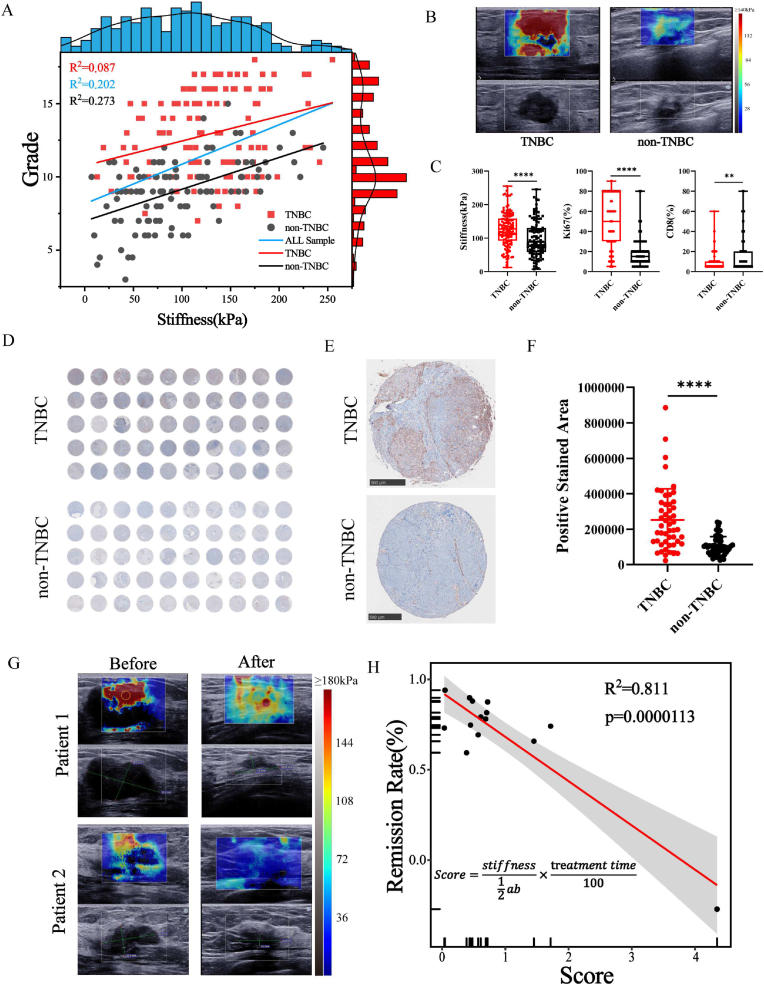
Clinical samples analysis. (A) Linear fitting analysis of patient SWE stiffness and pathological score in scatter plot. The data distribution was showed in histogram. (TNBC n = 131, non-TNBC n = 102). **(B)** Representative SWE images of TNBC and non-TNBC patients. **(C)** Boxplot of SWE stiffness value, Ki67 value, and CD8 value in 131 TNBC patients and 102 non-TNBC patients. The percentage values of Ki67 and CD8 were not continuous, but an integer multiple of 5%. **(D)** Immunohistochemical microarray panoramas of α-SMA expression in breast cancer tissue from 50 TNBC patients and 50 non-TNBC patients. **(E)** Representative images of α-SMA expression in the immunohistochemistry of TNBC and non-TNBC. Scale bar: 500 μm. **(F)** Quantitative results of α-SMA positive stained area in tissue microarray (each group n = 50). **(G**) Representative images of patients before and after neoadjuvant SWE imaging. The gray scale image contained measurements of tumor cross-section length and width. **(H)** Linear fitting analysis of patient SWE stiffness score and neoadjuvant remission rate in scatter plot. The score formula was showed in this graph (n = 14). Data expressed as mean ± SD.

The pathological indicators and tumor SWE stiffness of 233 patients were analyzed; 131 patients clinically diagnosed with TNBC and 102 patients diagnosed as non-TNBC were selected. The pathological results without any neoadjuvant therapy (excluding surgical resection) were recorded. First, we set up a system for scoring the pathological indicators Ki67 and CD8 by clinical pathologists (Fig. S1). We measured the tumor stiffness in the region of interest (ROI, diameter = 4 mm, the highest stiffness site was selected) through SWE imaging. We analyzed the correlation between the tumor stiffness value and pathological score (Ki67 score + CD8 score). An R^2^ value of 0.273 and 0.087 was obtained for the non-TNBC and TNBC groups, respectively; the R^2^ of correlation analysis for all samples was 0.202 (Fig. 1A). Coefficient of determination (R^2^) is the ratio of the sum of squares for regression to the sum of squares for total, R^2^ ≥ 0.25 is considered relevant. We speculated that both the measurement of pathological indicators and the acquisition of SWE imaging would be influenced by the subjectivity of individual physicians. However, a tendency for higher stiffness value to be associated with high Ki67 expression and low CD8 infiltration was observed (Fig. 1A). Further, we compared each pathological indicator between the two groups respectively. TNBC had higher tumor stiffness (p < 0.0001) and Ki67 expression (p < 0.0001), as well as lower CD8 infiltration (p < 0.01) compared with those in patients diagnosed as non-TNBC (Fig. 1B & C). These results suggested that TNBC tumors had worse TME than non-TNBC tumors in patients. Subsequently, α-SMA staining was performed on cancer tissue samples from 50 patients clinically diagnosed with TNBC and from 50 non-TNBC patients to assess the degree of fibrosis between them. As expected, TNBC tumors indeed had higher fibrosis than non-TNBC tumors (Fig. 1D, E, and 1F). It had been reported that fibroblasts mediate metastasis of tumors [37]. In addition, the age distribution of TNBC patients in clinical data analysis and tissue microarray was consistent with that of non-TNBC patients, which avoid the deviation in fibrosis degree and fibroblasts function caused by the age distribution difference between the two groups (Fig. S2). A large number of literatures [4,5,38] had indicated that CAFs play an important role in TME and were closely relevant to metastasis, drug resistance and immunosuppression. Based on the above results and literatures, TNBC tumors had a higher fibrosis degree and SWE stiffness than non-TNBC tumors. Higher fibrosis was associated with worse TME, and the TME was associated with drug resistance and immunosuppression [37,39]. Therefore, it seems feasible to use SWE imaging for predicting the effect of neoadjuvant. Interestingly, some patients with high SWE imaging stiffness and large tumor volume had significant neoadjuvant response efficacy. Some patients who have low stiffness and small volume of tumor had a weak neoadjuvant response efficacy (Fig. 1G). Further, instead of unidimensionally and directly associating tumor stiffness with neoadjuvant response, we should incorporate the length (a) and width (b) of SWE imaging section into a correlation formula. In 14 patients who were clinically diagnosed as TNBC and underwent neoadjuvant therapy, an evaluation formula was developed based on SWE imaging prior to neoadjuvant therapy and in the duration of the treatment. The remission rate was measured by the maximum cross-sectional area of the tumor before and after neoadjuvant therapy (Fig. S3). Since the treatment time also affected treatment outcome, the treatment time was also included in the evaluation system. Finally, we found that tumor stiffness was strongly correlated with the remission rate of the neoadjuvant, R-squared was 0.811, and p = 0.0000113, which proved that this correlation had a good reliability (Fig. 1H). Based on these results, they showed a good potential for predicting neoadjuvant through SWE imaging. α-SMA had been reported in the previous literature to be highly expressed in TNBC and luminal A breast cancer [3], but FAP expression in some kinds of breast cancer is low, so α-SMA as maker for CAFs is feasible. Based on the cogitations of clinical data, it is incompletely confirmed that SWE stiffness is related to CAFs functional status, or CAFs amount, or both of them. Therefore, we further explored and validated in mouse tumor model.

### 2.2 Characterization of biomimetic nanoparticles

**Fig. 2:**
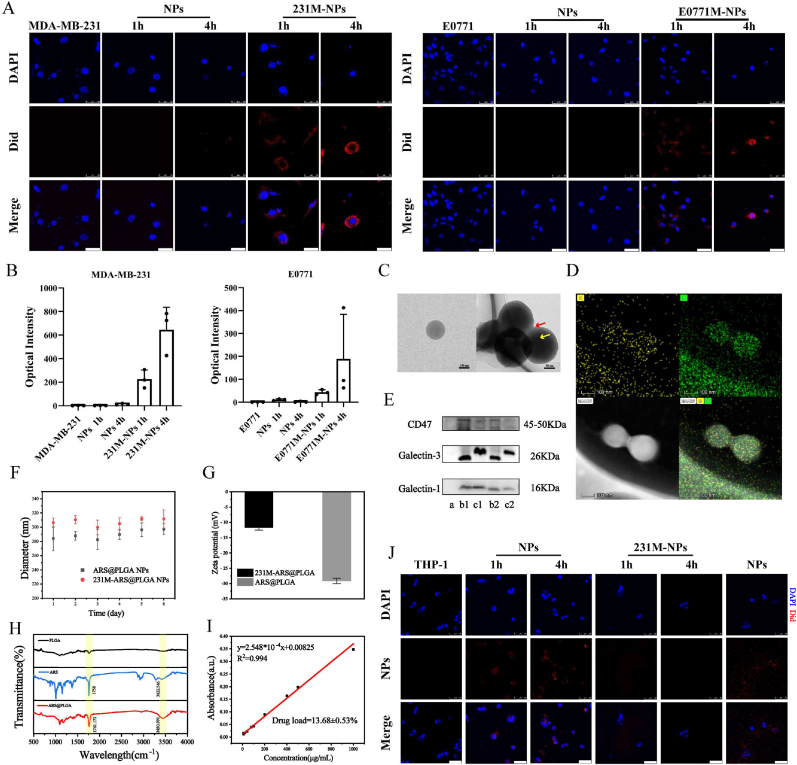
Characterization of biomimetic nanoparticles. **(A)** The CLSM images of homologous aggregation targeting experiments *in vitro* (cell: blue, NPs: red), scale bar: 50 μm. **(B)** Quantification results of optical intensity for each cell selected at random (n = 3). **(C)** FETEM images of AP NPs and 231M-AP NPs, the image on right side showed that the nanoparticles (yellow arrow) were coated with cell membrane (red arrow), scale bar: right: 50 nm, left: 100 nm. **(D)** FETEM elemental mapping of 231M-NPs, O (yellow), P (green), HAADF (gray), scale bar: 100 nm. **(E)** Western blot analysis of (a) NPs, (b1) 231 cell membrane, (c1) E0771 cell membrane, (b2) 231M-NPs, and (c2) E0771M-NPs. **(F)** Stability of NPs and 231M-NPs in water (n = 3). **(G)** Zeta potentials of bare nanoparticles and 231M-NPs (n = 3). **(H)** FTIR spectroscopy of PLGA, ARS, and AP NPs. **(I)** Concentration-Absorbance standard curve of ARS methanol solution at 222 nm (n = 3). **(J)** CLSM images of biomimetic nanoparticles (or NPs) immune escape *in vitro*. THP-1 (blue), 231M-NPs and NPs (red), scale bar: 50 μm. Data expressed as mean ± SD.

Artesunate (ARS) has a short half-life *in vivo*. Therefore, we designed engineered nanoparticles modified with 231 membrane (231M-NPs) and nanoparticles modified with E0771 membrane (E0771M-NPs) to improve the accumulation of ARS in different mouse tumor models. Galectin-1 and galectin-3 expression on the tumor cell membrane promotes the aggregation of homotypic tumor cells. To verify the homologous targeting ability of the nanoparticles, we incubated biomimetic nanoparticles (231M-NPs and E0771M-NPs) or unmodified nanoparticles with tumor cells for different incubation times. Few unmodified nanoparticles were endocytosed into the tumor cells after incubation for 4 h in confocal laser scanning microscope (CLSM) images; however, more biomimetic nanoparticles gathered around the tumor cells after 4 h (Fig. 2A). The homologous aggregation of E0771M-NPs to E0771 cells showed the same trend as the MDA-MB-231 cells (Fig. 2A). To verify whether biomimetic nanoparticles could target non-homologous cell lines, we incubated E0771M-NPs and 231M-NPs with T-47D cells respectively; the biomimetic nanoparticles had no obvious homologous targeting effect on T-47D cells (Fig. S4). These results indicated that the biomimetic nanoparticles have homologous targeting effect. Further, we quantified the amount of biomimetic nanoparticles endocytosed by cells through Image J, delineating cell edge and quantifying optical intensity in cells. Tumor cells were more selective for biomimetic nanoparticles than that in nanoparticles unmodified with cell membrane (Fig. 2B). The trend of homologous aggregation effect in flow cytometry were consistent with that in CLSM (Fig. S5). Therefore, we selected nanoparticles modified with MDA-MB-231 (231) cell membrane as the representative for characterization. Field emission transmission electron microscope (FETEM) imaging indicated that the 231M-ARS@PLGA nanoparticles (231M-AP NPs) were spherical and approximately 150 nm in diameter (Fig. 2C). Polydispersity index (PDI) also showed nanoparticles had good uniformity of particle size (Fig. S6). In addition, we performed an assessment of the homogeneity of different nanoparticle sizes and the homogeneity and stability of different biomimetic modification methods in preparation were also evaluated. Nanoparticles had better homogeneity between 200 and 300 nm, and the biomimetic nanoparticles had better homogeneity and stability when cell membrane: nanoparticles weight ratio was 1:1 (Fig. S7). The main elements of PLGA are C and O, and the membrane is mainly composed of the phospholipid bilayer. Elemental analysis of the 231M-NPs showed the presence of phosphorus and oxygen on the nanoparticles (Fig. 2D), proving that the membrane was successfully modified on the nanoparticles. Subsequently, proteins were extracted from the nanoparticles, cell membranes, and biomimetic nanoparticles. Western blot analysis indicated that CD47, galectin-1, and galectin-3 were detected on both the biomimetic nanoparticles and tumor cell membrane (Fig. 2E). The hydration particle size of the nanoparticles ranged from 200 nm to 340 nm, as measured using the particle size distribution analyzer; they showed good solution stability (Fig. 2F). As reported in literature [31], the tumor cell membrane surface potential is approximately −10 mV. The zeta potential on the nanoparticle surface changed from −29.17 ± 0.87 mV to −11.77 ± 0.76 mV (Fig. 2G). Fourier Transform Infrared (FT-IR) was used to determine the surface chemistry of PLGA, ARS, and ARS@PLGA NPs (AP NPs). ARS has a carboxyl peak at 1758 cm^−1^ and an O–H peak at 3422.546 cm^−1^; these peaks of AP NPs were slightly shifted to the high wave numbers of 1761.172 cm^−1^ and 3450.991 cm^−1^, respectively (Fig. 2H). Therefore, ARS did not react with PLGA, but was loaded into the nanoparticles in the free molecular state. Alkalization is deemed essential to determine the UV–Vis spectrum of ARS [40]. However, we dissolved different concentrations of ARS in methanol, and measured the absorption peak at 222 nm using a spectrophotometer. The standard curve of ARS concentration-absorbance could still be fitted, and the ARS loaded content in the AP NPs was determined to be 13.68 ± 0.53% (Fig. 2I). The expression of galectin-1 and galectin-3 on the tumor cell membrane induces homologous aggregation of tumor cells [33], and it confers biomimetic nanoparticles with tumor targeting ability. CD47 is highly expressed on normal cells and on tumor cells [35], which facilitates the immune escape of tumor cells *in vivo*. This also provides a basis for biomimetic nanoparticles to have good circulation *in vivo*. To verify the immune escape effect of the 231M-NPs, THP-1 cells were subjected to low serum treatment to induce starvation and differentiation into adherent macrophages. 231M-NPs loaded with Did fluorescent dye were co-cultured with THP-1 cells. Macrophages exhibited a stronger phagocytosis ability towards the non-biomimicking nanoparticles than to the 231M-NPs (Fig. 2J). Further, five cells from each group were randomly selected and the intracellular fluorescence intensity were quantified. The THP-1+NPs 4 h group showed higher fluorescence intensity than the other groups (Fig. S8). To ascertain that the fluorescence in the cell was not caused by dye leakage from the nanoparticles, we prepared Did-labeled nanoparticles with diameters ranging from 800 nm to 1 μm, and co-cultured them with THP-1 cells for 4 h. CLSM observations performed without removing the free nanoparticles showed that the cell membrane was not stained, indicating that the cellular fluorescence was originated from the nanoparticles that were phagocytosed by the cell (Fig. 2J).

### 2.3 Inhibition of fibroblasts mediated by ARS *In vitro*

**Fig. 3:**
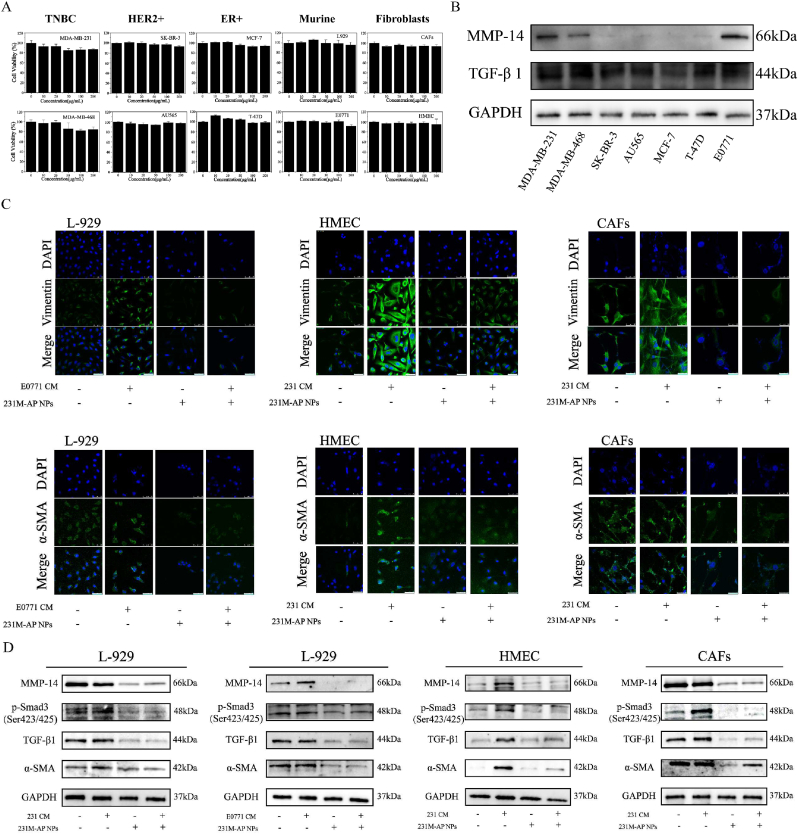
231M-AP NPs inhibition of CAFs function *in vitro*. (A) Cell toxicity of 231M-AP NPs on different cell lines with different nanoparticles concentrations for 24 h incubation (n = 4). **(B)** Western Blot analysis of promoting fibrosis ability in different breast cancer cell lines. **(C)** The CLSM images of 231M-AP NPs inhibiting fibroblast activation (cell: blue, maker protein: green), scale bar: 50 μm. (**D)** Western Blot analysis of 231M-AP NPs inhibiting fibrogenesis. Data expressed as mean ± SD.

Some studies have suggested that ARS can inhibit bcl-2 expression and induce apoptosis [41]. Here, we performed the CCK-8 assay on 6 human breast cancer cell lines (MDA-MB-231, MDA-MB-468, SK-BR-3, AU565, MCF-7, T-47D), a mouse TNBC cell line (E0771), and 3 fibroblasts (CAFs, L929, HMEC). 231M-AP NPs had no significant cytotoxicity on cells (Fig. 3A). Neither ARS nor AP NPs had any significant cytotoxicity (Fig. S9). We speculated that ARS did not directly inhibit tumor cells, but the anti-tumor effect in solid tumors was attributed to its anti-fibrosis effect. The formation of CAFs is largely induced by tumor cell-derived TGF-β1 [42,43]. MMP-14, a member of the matrix metalloproteinase family, promotes tumor invasion by degrading collagen [44]. Among the 7 breast cancer cell lines screened, TNBC cells (MDA-MB-231 and E0771) had higher CAF induction and invasion ability (Fig. 3B). Therefore, we selected those cells for the follow-up experiments. Subsequently, to validate the anti-fibrosis effect of ARS, we assessed the effects at the cellular level using immunofluorescence and Western blot assays. Vimentin expression is upregulated during epithelial mesenchymal transition (EMT) [45], which can be used as a maker for cells that develop EMT. Similarity, CAF-S4 enriched in TNBC was defined as FAP^negative^, α-SMA^high^, CD29^high^, FSP1^Low−Med^, FDGFR-β^Low-Med^, and CAV1^Neg-Low^ [3]. Further, it is expressed in metastatic lymph nodes. Therefore, Vimentin and α-SMA were used to evaluate the anti-fibrosis effect of ARS. All kinds of fibroblasts were incubated with MDA-MB-231 condition medium (231 CM) or E0771 condition medium (E0771 CM). Vimentin and α-SMA expression decreased significantly when fibroblasts were treated with 231M-AP NPs (Fig. 3C). 231M-AP NPs inhibited the expression of CAF-related proteins following co-incubation with the nanoparticles (Fig. 3D). Combined with the results from the CCK-8 assay, these results indicated that the effect of ARS on CAFs was more inhibitory than lethal.

### 2.4 Anti-tumor effect of artesunate *in vivo*

**Fig. 4:**
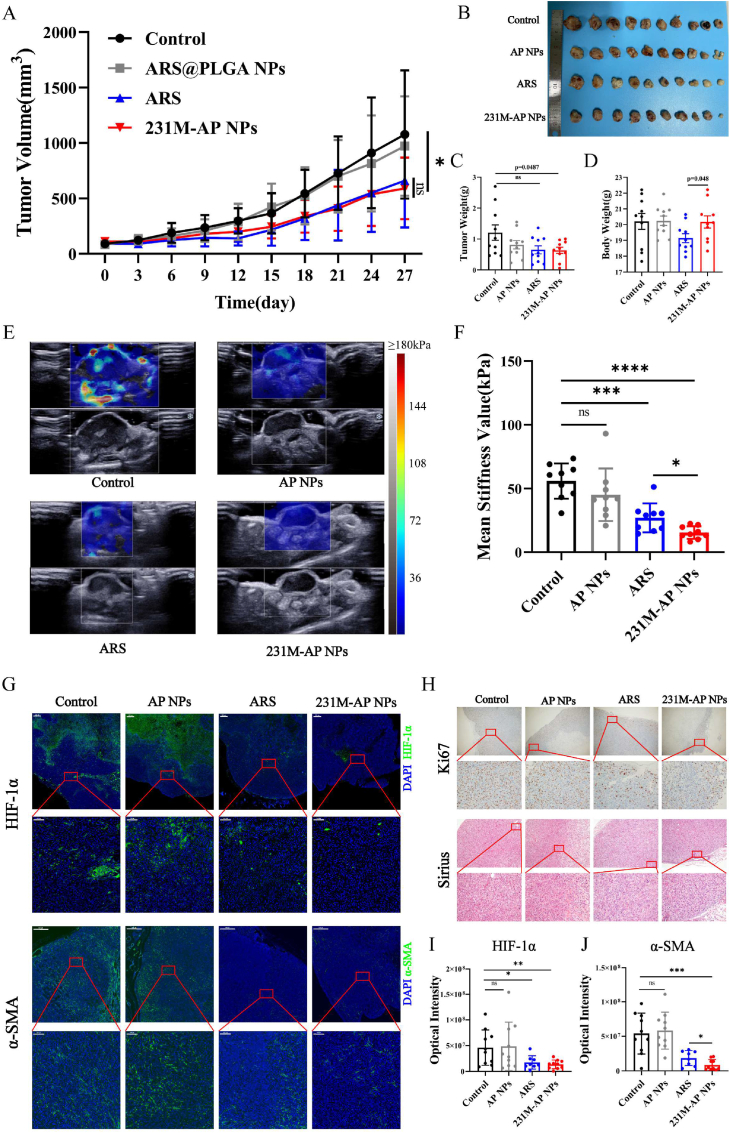
Artesunate mediated anti-tumor and SWE stiffness reversal effects *in vivo*. (A) Tumor volume curve in different groups during the 27-day monitoring period (n = 10). **(B)** Photograph of each tumor in different groups of mice on 27^th^ day of treatment. **(C)** Tumor weight of mice in different groups (n = 10). **(D)** Body weight of mice in different groups on 27^th^ day of treatment (n = 10). **(E)** Representative SWE images of tumors in different groups, each image is composed of pseudo-color image and gray-scale image. **(F)** Quantization results of ROI (diameter: 1 mm) in SWE images for tumors in different groups (n = 10, group ARS: n = 9). **(G)** Representative immunofluorescence images of HIF-1α (green) and α-SMA (green) staining of tumor tissue (blue) in different groups. Scale bar in low magnification: 500 μm, scale bar in high magnification: 100 μm. **(H)** The Sirius red staining and immunohistochemical images of tumor tissue in different groups under different magnifications (low magnification: 40 × , high magnification: 400 × ). **(I) & (J)** Quantification of HIF-1α and α-SMA biomarkers in tissue immunofluorescence sections (n = 10, group ARS: n = 8). Data expressed as mean ± SD.

To further explore the relationship between SWE stiffness and CAFs, MDA-MB-231 xenograft tumor bearing mice (n = 40) were randomly divided into 4 groups: control, ARS, AP NPs, and 231M-AP NPs. The ARS group and 231M-AP NPs group showed only a weak antitumor effect after 27 days of treatment (once injection/3 days) (Fig. 4A and B). The change trend in tumor weight was consistent with that in tumor volume (Fig. 4C). These results were consistent with that from the CCK-8 assay *in vitro*. However, considering the body weight change of mice in different groups, the 231M-AP NPs showed lower side effect than ARS (Fig. 4D and S10). On the 25^th^ day, SWE imaging was used to evaluate the tumors in all groups. The tumor stiffness in the 231M-AP NPs and ARS groups were lower than that in the control and AP NPs groups (Fig. 4E & F). The biomimetic nanoparticles (231M-AP NPs) were better at reducing tumor stiffness than ARS as well as had lower toxicity than ARS.

Although the ARS and 231M-AP NPs groups did not show satisfactory anti-tumor effects, but as expected, they reversed tumor stiffness and altered the characteristics of SWE imaging. SWE stiffness has been reported to be positively correlated with tumor hypoxia [13]. By staining the tumor tissue sections, we observed that ARS and 231M-AP NPs decreased HIF-1α expression and significantly attenuated hypoxia in solid tumors (Fig. 4G). In addition, the expression of α-SMA in tumor tissues was significantly decreased. Based on the results of the CCK-8 assay and immunohistochemistry, we concluded that the ARS and 231M-AP NPs could inhibit CAF function to alter the tumor microenvironment, which was further confirmed through Sirius staining (Fig. 4H). We hypothesized that TME remodeling (inhibition of CAFs function) reduced the proliferation of tumor cells (Fig. 4H), causing the difference in tumor volume between the 231M-AP NPs and Control groups. Therefore, 231M-AP NPs acts as a tumor soil-loosening agent for solid tumors through TME remodeling. We stained the immunofluorescence sections of all tumor samples, and quantified the fluorescence intensity corresponding to HIF-1α and α-SMA using Image J. HIF-1α and α-SMA were significantly downregulated in the ARS and 231M-AP NPs groups (Fig. 4I & J). The H&E staining of the organ sections of mice indicated a large number of inflammatory cells in the livers from the AP NPs group. This could be attributed to the immunological rejection induced by nanoparticles *in vivo*. The extent of liver inflammation was relatively mild in the 231M-AP NPs group compared to that in the other groups (Fig. S11). Based on the above results, 231M-AP NPs group achieved better results in reversing tumor stiffness and remodeling TME than ARS group, suggesting that biomimetic nanoparticles had better drug delivery ability. In the following experiments, the biomimetic nanoparticles would help us more significantly compared the SWE imaging changes caused by inhibiting CAFs function.

### 2.5 *In vivo* artesunate-mediated sensitization for chemotherapy

CAFs in the TME play an important role in drug resistance and invasion of tumors [5,46]. The biomimetic nanoparticles act as a “soil loosening agent” for solid tumors to inhibit the function of CAFs and remodel the TME. Paclitaxel (PTX), the first-line drug in breast cancer treatment, was used to validate whether TME remodeling could amplify the antitumor effect of chemotherapy drugs. We designed 231M-ARS/PTX@PLGA nanoparticles (231M-APP NPs) to validate this hypothesis. First, the 231M-APP NPs were characterized. The additional loading of PTX did not change the physical characteristics of the nanoparticles significantly. FT-IR analysis of ARS/PTX@PLGA NPs (APP NPs) (Fig. S12) showed that the amide group of paclitaxel (PTX) has a carbonyl peak at 1646.91 cm^−1^, a ketone carbonyl absorption peak at 1729.35 cm^−1^, and an extensive hydroxyl stretching vibration peak at 3439.42 cm^−1^. The corresponding characteristic peaks were found at 1645 cm^−1^, 1759.24 cm^−1^, and 3305.87 cm^−1^. The standard curve of PTX concentration-absorbance was also fitted similarly (Fig. S13). To evaluate the content of ARS and PTX in NPs, we dissolved ARS and PTX in methanol at 1:1 ratio. PTX influenced the absorbance of ARS at 222 nm, but ARS did not affect the absorbance of PTX at 273 nm. The slope of the standard curve for the methanol solution of PTX is consistent with that of the PTX and ARS mixture. The drug-loaded content of APP NPs was 14.96 ± 4.57% (ARS) and 6.25 ± 0.03% (PTX), respectively (Fig. S14). Although ARS had no lethal effect on tumor cells, we noticed that the combination of ARS and PTX had an excellent antitumor effect than PTX alone (Fig. S15A). 231M-APP NPs also indicate that ARS could amplify the antitumor effect of PTX, and the antitumor effect was positively correlated with incubation time (Fig. S15B).

Therefore, 40 MDA-MB-231 xenograft tumor bearing mice were randomly divided into 4 groups: control, PTX, PTX + ARS, and 231M-APP NPs. After 21 days of treatment, both the PTX + ARS and 231M-APP NPs groups showed good therapeutic effects, especially in the 231M-APP NPs group (Fig. S16A, S16B and S17). We also noticed that 231M-APP NPs induced less side effect than pure drug combination, according to body weight change results in mice (Figs. S18 and S19). As expected, ARS decreased α-SMA expression and collagen secretion in tumors. The expression of α-SMA and collagen might be due to CAFs activation caused by tumor traumatic stress (Fig. S20A, S20B & S20C). The H&E stained sections of mouse viscera indicated that the hepatic reaction caused by PTX was more severe than that induced when administered with 231M-APP NPs. A lower inflammatory response means fewer phagocytosed by white blood cells, so biomimetic nanoparticles might have better circulation ability *in vivo* (Fig. S21).

### 2.6 *In vivo* E0771M-AP NPs-mediated sensitization for immunotherapy

**Fig. 5:**
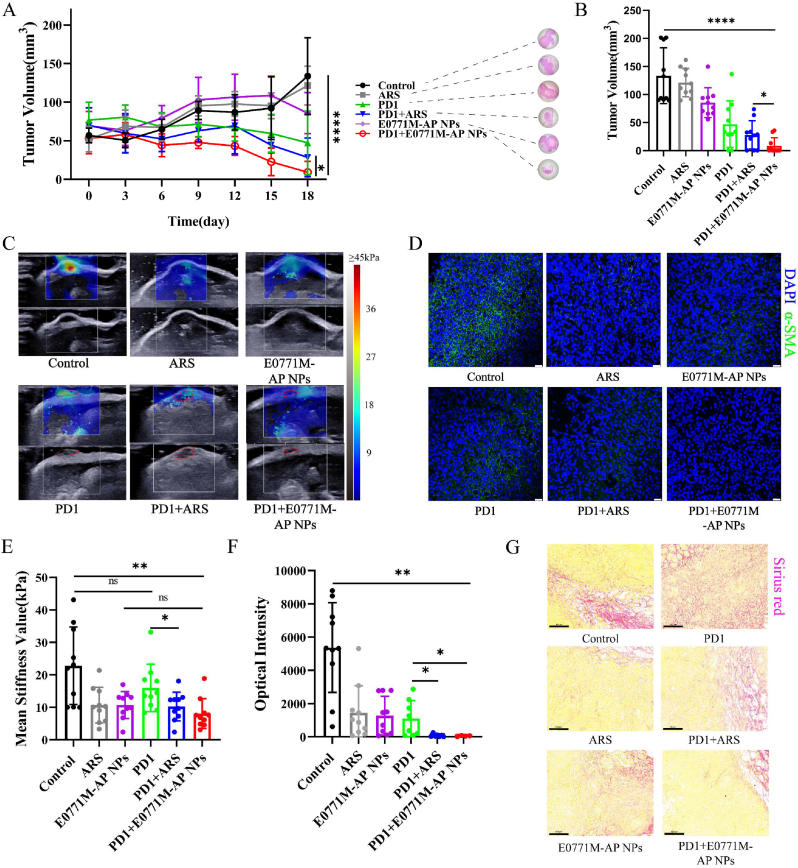
E0771M-AP NPs mediated SWE stiffness reversal through inhibiting CAFs function status in tumor. (A) Tumor volume curve in different groups during the 18-day monitoring period. Representative images of H&E sections of mouse tumor from different groups were used to compare the therapeutic effects of different groups (n = 10). **(B)** Tumor volume of mice in different groups at 18^th^ day (n = 10). **(C)** Representative SWE images of tumors in different groups. The red curve marked as tumor contour. **(D)** Representative tissue immunofluorescence images of the expression of α-SMA (green) in tumor tissue from different mouse groups, scale bar: 25 μm. **(E)** Quantization results of ROI (diameter: 1 mm) of tumor SWE images in different groups (n = 10). **(F)** Image J quantization results of α-SMA fluorescence in immunofluorescence. (Group: Control, ARS, E0771M-AP NPs, n = 10; group: PD1, n = 8; group: ARS + PD1, n = 7; group: PD1+E0771M-AP NPs, n = 4). **(G)** Representative microscope images of Sirius red stained tumor tissues in various groups (scale bar = 100 μm). Data expressed as mean ± SD.

Nude mice, as immunodeficient mice, xenograft tumor bearing mice could not completely mimic the TME of patients. In addition, we selected a mouse model with TME more similar to human TME to further confirm the correlation between SWE imaging stiffness and CAFs function. Generally, tumor hypoxia is closely related to immunosuppression in the TME [17,47]. The above-mentioned results demonstrate that ARS can attenuate hypoxia in solid tumors. Although E0771 and MDA-MB-231 are both TNBC cells, but they did not belong to the same genus. We modified nanoparticles with E0771 membrane in following experiments to increase the compatibility and homologous aggregation of nanoparticles in mice. Therefore, we examined the effect of a PD1 inhibitor in combination with the E0771M-AP NPs (tumor soil loosening agent) in treating E0771 tumor bearing mice. Sixty C57/B6J mice were randomly divided into 6 groups: control, ARS, PD1, PD1+ARS, E0771M-AP NPs, and PD1+E0771M-AP NPs. After 18 days of treatment (Fig. S22), the PD1+E0771M-AP NPs group showed significant therapeutic effects compared to the PD1, PD1+ARS, and E0771M-AP NPs groups (Fig. 5A and B, and S23). Further, mice treated with biomimetic nanodrugs (E0771M-AP NPs) experienced lower toxicity compared to mice treated with the drugs (ARS) directly (Figs. S24A and S24B). We then performed SWE imaging on the tumors. As expected, the tumors treated with ARS or the E0771M-AP NPs showed varying degrees of stiffness reduction (Fig. 5C), reinforcing our view that CAFs functional status also affected tumor stiffness. Tumor tissue sections also showed α-SMA expression decreased with decreased tumor SWE stiffness (Fig. 5D). The quantitative results of tumor SWE imaging stiffness showed that PD1 inhibitor alone did not reduce tumor stiffness significantly. Tumor SWE imaging stiffness would be reduced when CAFs function was inhibited (Fig. 5E). In Fig. 5F, α-SMA expression was significantly inhibited in tumors which were reduced in SWE imaging stiffness. Further, Sirius red stained showed that there were more thick collagen fibers in Control group, while the collagen fibers in PD1 group were small and disordered. Other groups showed that ARS or the tumor soil loosening agent we prepared reduced collagen production (Fig. 5G). PD1 inhibitor-mediated tumor traumatic stress resulted in CAFs activation, but SWE imaging stiffness did not increase significantly. This phenomenon was consistent with Fig. 1G. Therefore, we believed that SWE imaging stiffness is jointly determined by the degree of fibrosis in tumor and the function status of CAFs.

**Fig. 6:**
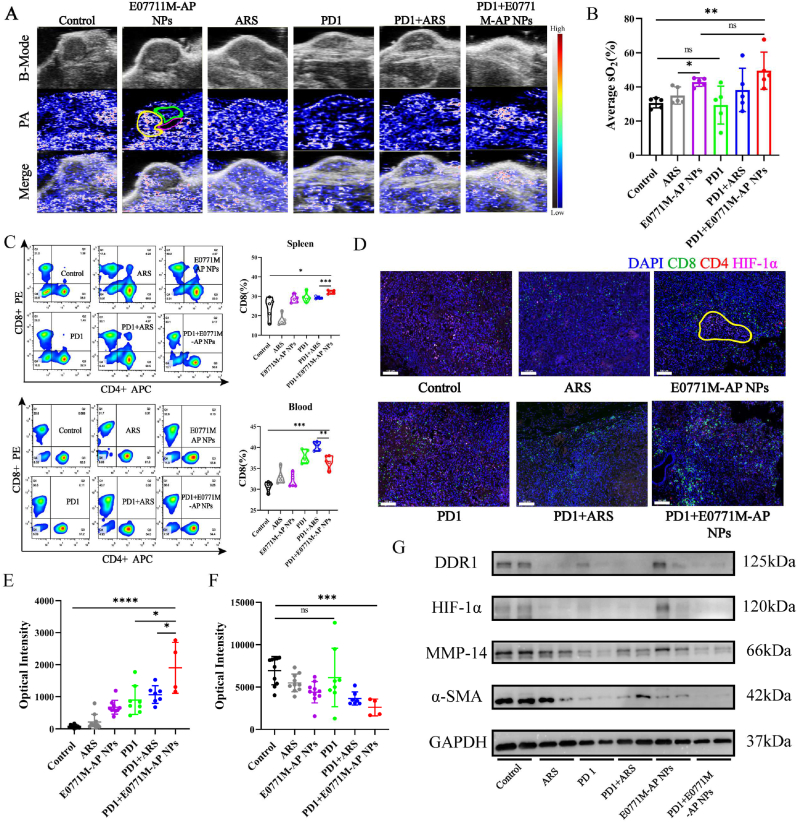
E0771M-AP NPs mediated tumor microenvironment remodeling through attenuating hypoxia. (A) Representative images of photoacoustic imaging to assess blood oxygen level within tumor. The yellow curve marked non-hypoxic area, the green curve marked as hypoxic area, and the red curve marked as necrotic area. **(B)** Quantization results of ROI (along the margin of tumor) of tumor photoacoustic images in different groups (n = 5). **(C)** Flow cytometry was used to analyze the proportion of CD8^+^ T (PE) cells and CD4^+^ T (APC) cells in immune cells (FITC anti-CD3) of different body parts (n = 5). **(D)** Representative tissue immunofluorescence images of the expression of CD8 (green), CD4 (red), and HIF-1α (pink) in tumor tissue of different mouse groups. The yellow curve showed the region of HIF-1α high expression and CD8 low expression, scale bar: 100 μm **(E&F)** Image J quantization results of CD8, HIF-1α fluorescence in immunofluorescence from left to right successively. (Group: Control, ARS, E0771M-AP NPs, n = 10; group: PD1, n = 8; group: ARS + PD1, n = 7; group: PD1+E0771M-AP NPs, n = 4). **(G)** Western Blot analysis of the DDR1, HIF-1α, MMP-14, α-SMA expression on tumor tissue in different groups (n = 2). Data are represented as mean ± SD.

To investigate the mechanism of attenuating hypoxia in TME with E0771M-AP NPs, we performed photoacoustic (PA) blood oxygen imaging on mouse tumor. Interestingly, the tumors treated with ARS or the E0771M-AP NPs showed an increased trend of blood oxygen in the tumor (Fig. 6A & B). In Fig. 6A, the area in the yellow circle represents the oxygen-rich area, the green circle represents the potential oxygen-barren area, and the red circle represents the necrotic area. We further hypothesized that the drug may increase vascular remodeling in the solid tumor, improving the proportion of normal blood vessels in the tumor. This could be one of the reasons for the attenuated hypoxia in the treated tumors. Immunosuppression in TME is often associated with hypoxia in TME. Therefore, we analyzed the CD8^+^ T cell infiltration in the spleen and blood of different groups using flow cytometry, and T cell infiltration in the tumor using tissue immunofluorescence. The results indicated that PD1 inhibitor activated the spleen to produce more T cells, and that the E0771M-AP NPs combined with the PD1 inhibitor could recruit more CD8^+^ T cells into the tumor, which slightly decreased the percentage of CD8^+^ T cells in the blood (Fig. 6C). Artesunate was reported by earlier literatures [[48], [49], [50]] that could increase proportion of CD8^+^ T cells *in vivo*, it was also shown in Fig. 6C. In addition, there was no infiltration of CD4^+^ and CD8^+^ T cells in the area with high HIF-1α expression (the area marked by the yellow circle), verifying that hypoxia is associated with immunosuppression in the tumor (Fig. 6D). We stained all tumor slices and quantified the fluorescence intensity of randomly selected areas under the CLSM. The quantification results showed that E0771M-AP NPs increased the infiltration of CD8^+^ T cells in the tumor (Fig. 6E) and attenuated the hypoxic in the tumor (Fig. 6F). ARS and E0771M-AP NPs enhanced PD1 inhibitor antitumor effect through attenuating hypoxia in TME. To assess treatment effect, breast pad sections were made and observed under the microscope after H&E staining. Breast pads were selected from the pads marked by yellow box in Fig. S25. In Fig. S25, there were only three breast pads had little residual tumor tissue in PD1+E0771M-AP NPs group. The anti-tumor effect was higher in PD1+E0771M-AP NPs group than in PD1 group and PD1+ARS groups. In addition, we sectioned the heart, liver, and lungs, because they are organs with high distribution of fibers. The H&E staining showed no significant damage to the organs (Fig. S26). E0771M-AP NPs could inhibit tumor proliferation as shown from immunohistochemical analysis of TUNEL in tumor tissue (Fig. S27A). Immunohistochemistry showed that Ki67 expression in tumors treated with ARS or biomimetic nanoparticles was also decreased (Fig. S27B). DDR1 is directly associated with tumor cell immune escape in TNBC [4,51], and with fibrosis and collagen synthesis [4]. The tumor soil loosening agent attenuated the tumor hypoxic state and inhibited the functional state of CAFs; we randomly selected two tumor tissues from each group of mice and performed Western blot analysis for further verification. The treatment with the tumor soil loosening agent combined with the PD1 inhibitor significantly decreased the expression of DDR1, HIF-1α, MMP-14, and α-SMA (Fig. 6G).

### 2.7 Distribution of the biomimetic nanoparticles *in vivo*

**Fig. 7:**
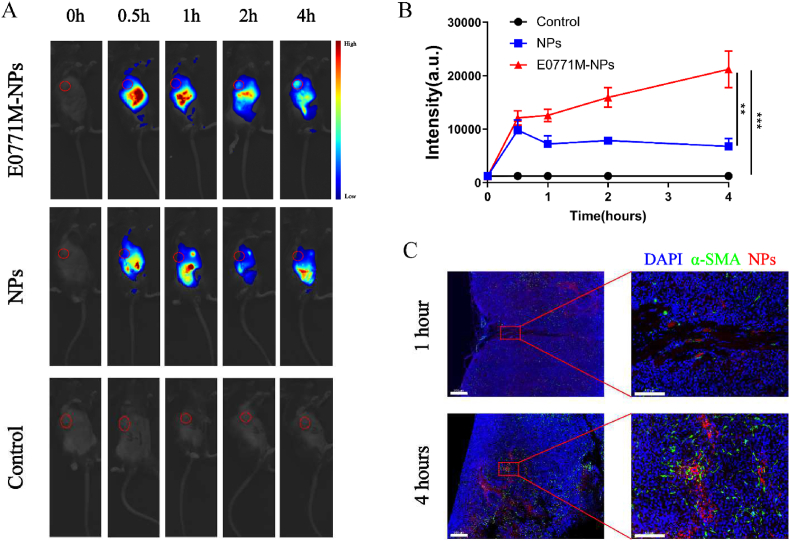
Distribution of the biomimetic nanoparticles *in vivo*. (A–B) Representative images and quantification bioluminescence of breast tumor mice model in different groups (n = 5). **(C)** Representative immunofluorescence images of NPs (red) accumulation in tumor tissue (blue) through tumor blood vessels (green), scale bar in low magnification: 200 μm, scale bar in high magnification: 100 μm. Data expressed as mean ± SD.

To observe the metabolism of biomimetic nanoparticles (E0771M-NPs) *in vivo*, we loaded cypate, a derivative of indocyanine green (ICG), into the nanoparticles and prepared seven concentrations gradients (200, 100, 50, 20, 10, 5, and 0 μg/mL) in a microcentrifuge tube; we then observed the relationship between the NP concentration and fluorescence intensity under a bioluminescence detection instrument. A linear relationship was found between the two, but only when the concentration was below 50 μg/mL. When the concentration reached more than 100 μg/mL, cypate also demonstrated aggregation-caused quenching similar to ICG [52] (Figs. S28A and S28B). The metabolic behavior of nanoparticles in mice was observed after injecting them via the tail vein (Dosage: 4 mg/mL, 200 μL). Compared with unmodified nanoparticles, E0771M-NPs loaded with cypate showed a more obvious tumor targeting effect. In NPs group, the most of nanoparticles had been metabolized into the kidney at 0.5 h, and then metabolized into the bladder from 1 h to 4 h. In contrast, the most of biomimetic nanoparticles circulated in the liver, while only a small part of them concentrated in kidney and bladder, which indicating that the most of biomimetic nanoparticles were still circulating *in vivo* (liver and kidney are the two main organs with the most abundant blood supply in the body). The fluorescence curve of the tumor showed that the accumulation of nanodrugs in tumor gradually increased with circulated time going; And the difference between E0771M-NPs group and NPs group was significant (Fig. 7A & B). In the sixth hour after intravenous injection of the nanoparticles, mice were randomly selected for dissection, and their organs and tumors were collected for bioluminescence analysis. Fig. S29 showed that the E0771M-NPs still showed accumulation in the tumor at the sixth hour, but most remained in the liver, spleen and kidney. To further examine the accumulation of nanoparticles in the tumor, we used nanoparticles loaded with Did dye and obtained tumor sections at the 1^st^ hour and 4^th^ hour after tail vein injection. α-SMA was used to locate the blood vessels. As shown in Fig. 7C and S30, after the 1^st^ hour of injection, most of the nanoparticles remained in the blood vessels, but at the 4^th^ hour after injection, more nanoparticles accumulated in the tumor stroma. Nanoparticles enter solid tumors mostly through the endocytosis of endothelial cells; almost 4 h is needed to complete the transendothelial transport (Fig. 7C and S30), which were consistent with that from the earlier report [53].

### 2.8 Single cell sequencing to determine the mechanism of CAFs influencing SWE stiffness

In order to explore the relationship between tumor SWE stiffness and CAFs, we selected 2 mice each from the Control, PD1, and PD1+E0771M-AP NPs groups. We performed single-cell sequencing for each tumor. The cell quality control (QC) standard was nCount RNA ≥ 1000, nFeature RNA ≥ 200, log_10_GenesPerUMI >0.7, and mitochondrial-derived UMI counts <15%.

**Fig. 8:**
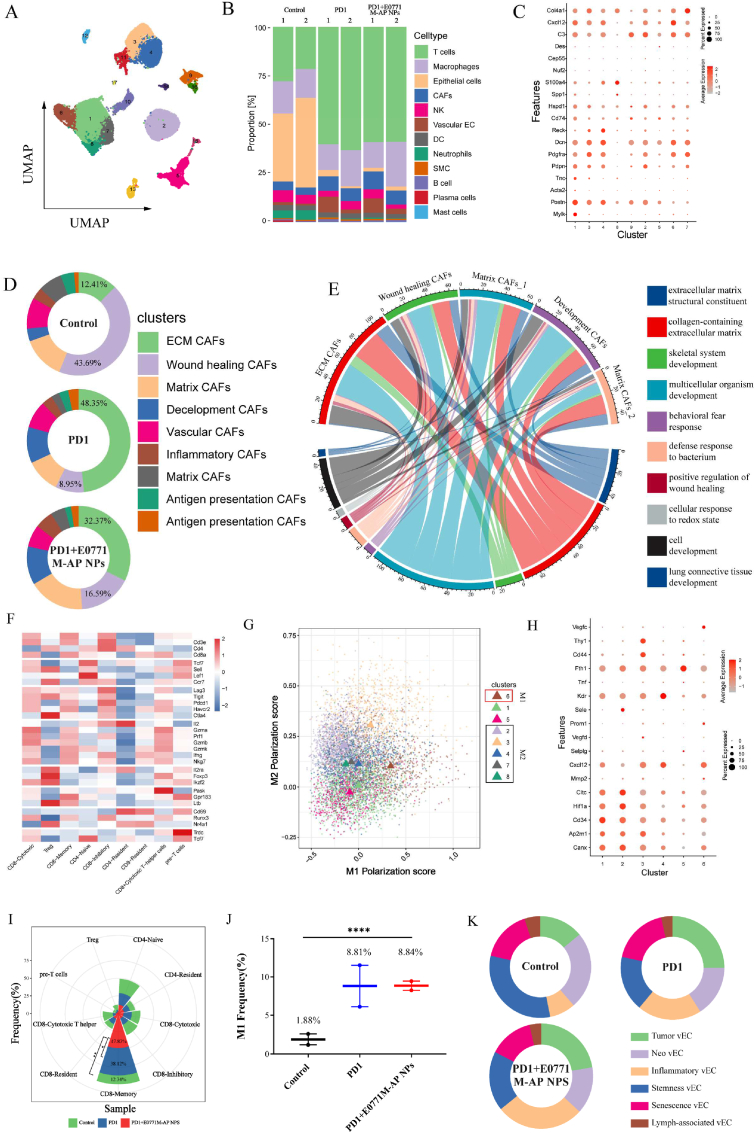
Single cell sequencing analysis of mouse tumors. (A) An uniform manifold approximation and projection (UMAP) view of 39968 single cells, color-coded by assigned cell type. **(B)** Proportion of each cell type in individuals. **(C)** Dot plot showing gene expression pattern of each cluster, which dot size and color indicate the fraction of expressing cells and average scaled expression value, respectively. **(D)** Pie chart of different CAFs subtypes proportion in different groups. **(E)** Chord diagram based on the relationship between gene expression of different CAFs subtypes and specific pathways. **(F)** The heatmap of makers in different clusters, which can help identify cell type. **(G)** Scatter plot of polarization score of macrophages in each cluster. The red box is M1 macrophages, the black box is M2 macrophages. **(H)** Dot plot showing gene expression pattern of each vascular endothelial cell cluster, which dot size and color indicate the fraction of expressing cells and average scaled expression value, respectively. **(I)** The proportion of different T cell subtypes from T cells in rose chart (n = 2). **(J)** Scatter diagram of M1 macrophages proportion in different groups (n = 2). **(K)** Pie chart of vEC subtypes proportion in different groups. Data expressed as mean ± SD.

Based on the same gene set, two-dimensional projection by uniform manifold approximation and projection (UMAP) grouped the cells distinctly into seventeen groups (Fig. 8A). We classified all cells according to the specific Maker gene into 12 clusters (Fig. 8B and S31). These cell types were mainly divided into: T cells, macrophages, epithelial cells, CAFs, natural killer cells (NK), vascular endothelial cell (vEC), dendritic cells (DC), neutrophils, smooth muscle cell (SMC), B cells, plasma cells, and mast cells. Vascular endothelial cells (vECs) increased in the PD1+E0771M-AP NPs group compared to that in the other two groups (Fig. 8B). This was verified by the results in Fig. 6A, where artesunate increased the blood supply to alleviate intratumor hypoxia. Cancer associated fibroblast cells were identified through the classic Maker gene (*Mmp2*, *Apod*, *Dcn*, and *Col1a1*) in all cells. CAF cells were clustered by gene expression and divided into 9 clusters (Fig. S32). We authenticated cluster 1 expressing the *TNC* gene as extracellular matrix CAFs [54,55](ECM CAFs), which mainly secreted extracellular matrix. *Pdgfra* and *Pdpn* are important Maker gene of matrix CAFs [54] (mCAFs), which were expressed in cluster 2, cluster 3, and cluster 7. Further, *Cep55* and *Nuf2* expression in cluster 2 implied that this cluster was an active and proliferative CAF cluster [56]. Therefore, we defined it as wound healing CAFs. Wound healing CAFs were also major producers of extracellular matrix due to their active status. Cluster 5 was defined as development CAFs (dCAFs), based on their expression of the *Reck* gene [56]. The signature maker gene of dCAF is *Scrg1*. However, we could not identify the expression of this gene in any of the clusters. *Reck* gene was also a dCAFs maker gene. dCAF is mainly involved in cell differentiation in solid tumors. Cluster 5 expressing *acta2* and *Des* was defined as vascular CAFs (vCAFs), which are mainly involved in angiogenesis. CAFs with high *Cxcl12* and *C3* expression level were defined as inflammatory CAFs; they have immunomodulatory role in the TME. *CD74* and *S100a4* were expressed in clusters 8 and 9, respectively; they were defined as antigen presenting CAFs (Fig. 8C). The total proportion of ECM CAFs (32.37%) and wound healing CAFs (16.59%) among all types of CAFs were lower in the group treated with E0771M-AP NPs than that in the other groups (PD1 group: 48.35%, 8.95%; Control group: 12.41%, 43.69%) (Fig. 8D). Several pathways we interested were selected. We then observed the number of genes that could be enriched into these pathways in all the expressed genes of each kind CAFs, which could be evaluated the association between these pathways and different types of CAFs. ECM CAFs and wound healing CAFs mainly influenced extracellular matrix synthesis (Fig. 8E); they were decreased in PD1+E0771M-AP NPs group. This evidence along with the results from Fig. 4, Fig. 5C & 5D strongly supports our hypothesis. Therefore, tumor stiffness is related to the content and functional status of CAFs.

In addition, we analyzed T cells, macrophages, and vEC to observe the effect of E0771M-AP NPs on TME remodeling. We used *CD3d* and *CD3g* as Maker gene to recognize T cells; a heat map was drawn based on the gene expression in these cells. The highly expressed genes (Fig. 8F) were used to recognize the subtypes of T cells, and for the two-dimensional projection using UMAP (Fig. S33). T cells were divided into 10 clusters (CD8^+^ cytotoxic T cell, Treg, CD8^+^memory T cells, CD4^+^ naive T cells, CD8^+^ inhibitory T cells, CD4^+^ resident T cells, CD8^+^ resident T cells, CD8^+^ cytotoxic T helper cells, and pre-T cells). The E0771M-AP NPs enhanced the infiltration of CD8^+^memory T cells in the tumor (Figs. S34 and 8I), which was consistent with the results in Fig. 6D. In order to evaluate the TME remodeling in multiple dimensions, we assessed the macrophages polarization score for the six macrophage clusters. Cluster 6 was identified as M1 macrophages, while cluster 2, cluster 3, cluster 4, cluster 7, and cluster 8 were identified as M2 macrophages, and the rest were identified as non-M1 and non-M2 macrophages (Fig. 8G). Compared to that in the control group, the proportion of M1 macrophages was increased in the PD1 and PD1+E0771M-AP NPs groups (Fig. 8J and S35). The vascular endothelial cells (vEC) were identified based on earlier reports and the gene expression of EC in all the groups (Fig. S36) [57]. *CD34* is the classical maker gene for tumor vessels; in addition, *Cltc*, *Canx*, and *Ap2m1* were highly expressed in tumor vessels [58]; these vessels could transport nanoparticles into tumor stroma. We identified cluster 1 as tumor endothelial cells. Cluster 2 was similar to cluster 1 in gene expression; however, the high expression of *mmp2* and *Hif1a* indicated that it was tumor neovascularization endothelial cell (tumor neo vEC) [59,60]. *Vegfd* is an important maker gene for the lymphoendothelial cell [61,62]; *cxcl12* and *selplg* are inflammation related factors in lymphocyte chemotaxis or transport. Therefore, cluster 3 was identified as inflammatory EC. Cluster 4 was identified as stemness EC based on the expression of *Kdr*, *Prom1*, and *Sele*. Cluster 5 was identified as senescence EC based on the expression of *Fth1* and *Tnf*. Cluster 6 was identified as lymph-associated EC based on the expression of *Vegfc*, *Il6*, and *CD44* (Fig. 8K). ARS increased the proportion of inflammatory EC (Fig. 8K). We hypothesized that blood vessels containing inflammatory endothelial cells could aid in the infiltration of immune cells into the tumor and the increased blood oxygen supply to attenuate hypoxia.

Therefore, through single-cell sequencing, we demonstrated that the E0771M-AP NPs could remodel the TME and tumor SWE stiffness was closely related to the functional status of CAFs. TME remodeling was associated with increased CD8^+^memory T cells infiltration, decreased non-M2 macrophage proportion, and increased inflammatory endothelial cells in the tumor. Tumor SWE imaging stiffness was mainly decided by ECM CAFs and wound healing CAFs.

### 2.9 GO enrichment analysis of gene pathway

**Fig. 9:**
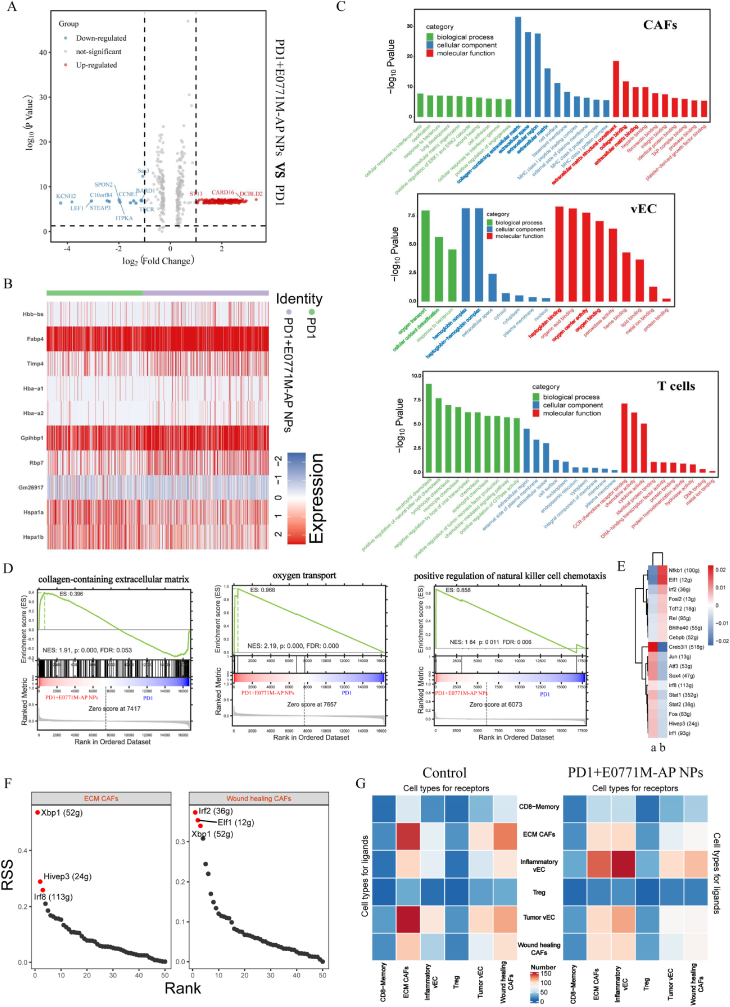
GO enrichment analysis of gene pathway between PD1+E0771M-AP NPs group and PD1 group. (A) Volcanic map of differential genes in cancer-associated fibroblasts between PD1 group and PD1+E0771M-AP NPs group. Typical genes were marked in the diagram. The threshold values were p < 0.05 and |log_2_FC| >1. **(B)** Heatmap of top 20 differential genes in vascular endothelial cells between PD1 group and PD1+E0771M-AP NPs group. **(C)** Gene pathway enrichment analysis based on gene differences between cancer associated fibroblasts, vascular endothelial cell, and T cells in PD1+E0771M-AP NPs group and PD1 group. Key pathways were marker in bold. **(D)** GSEA analysis between group PD1+E0771M-AP NPs and group PD1 based on differential expression genes. The graph was consisted of line chart of gene enrichment score and rank distribution of all genes. **(E)** The heat map of the most activity regulons in ECM CAFs and wound healing CAFs (a: ECM CAFs; b: wound healing CAFs). **(F)** The RSS ranking plot of the regulon with the highest specific correlation of ECM CAFs and Wound healing CAFs. The red dots are the top 3 regulons with highest score. **(G)** The heat map of ligand-receptor interaction pair number between different cell types in Control group and PD1 + E0771M-AP NPs group.

In order to clarify the specific pathway of E0771M-AP NPs reversing tumor SWE imaging stiffness and attenuating TME hypoxia. Differential gene enrichment analysis was performed on CAFs in PD1 group and PD1+E0771M-AP NPs group. We analyzed and compared the genes of CAFs between the two groups, and screened the differential genes based on the threshold values. Among these differential genes, we found that *Bard1*, *Spon2*, *Saa3*, and *Tecr* genes were significantly inhibited. Further, *St13*, *Card16*, and *Dcbld2* were significantly upregulated (Fig. 9A). Similarly, we further analyzed the differential genes in vascular endothelial cells between the two groups (Fig. 9B). GO enrichment analysis were performed by the above analysis. As shown in Fig. 9C, pathway changes of CAFs in these groups mainly focused on extracellular matrix. E0771M-AP NPs could reduce extracellular matrix and collagen secretion through inhibiting CAFs function. We selected three pathways collagen-containing extracellular matrix, oxygen transport and positively regulation of natural killer cell chemotaxis to perform gene set enrichment analysis (GSEA). The gene sets related to collagen-containing extracellular matrix pathway in PD1+E0771M-AP NPs group was higher than PD1 group, which indicating that E0771M-AP NPs mainly affected this pathway. But oxygen transport in tumor and positively regulation of natural killer cell chemotaxis was enhanced in PD1+E0771M-AP NPs group (Fig. 9D). This evidence undoubtedly reinforced the above results, suggesting that tumor SWE imaging stiffness is influenced by extracellular matrix secreted by CAFs. Further, we performed gene pathway enrichment analysis on vascular endothelial cells. After remodeling tumor microenvironment, the vascular endothelial cell ability to oxygen transport as well as the affinity for hemoglobin complex and oxygen carrier activity were enhanced. These results attenuated hypoxia in tumor microenvironment. The reason why the PD1+E0771M-AP NPs group had more CD8^+^memory T cells in tumor that the TME contained more chemokines after remodeling TME. In addition, favorable tumor microenvironment also enhanced the positive regulation of immune cells in it. Tumor microenvironment remodeling mediated by E0771M-AP NPs was reflected in reducing extracellular matrix secretion through inhibiting CAFs function and improving T cells activity through enhancing CAFs mediated immune response. In addition, we analyzed the related regulons of ECM CAFs and wound healing CAFs, and screened out the highest specificity score and activity score regulons of the two CAFs. Cell types are generally maintained by coordinated interactions between transcription factors and their corresponding target genes. In regulon activity score (RAS), ECM CAFs showed STAT pathway was closely to it. STAT1 and STAT3 played opposite role in tumor [63,64]. As STAT3 inhibitor, artesunate could be considered to specifically inhibited ECM CAFs. Wound healing CAFs was more related to *nfkb1*, mediating the occurrence of chronic inflammation in tumors and promoting tumor invasion and growth [65] (Fig. 9E). *Xbp1*, *hivep3*, *lrf8*, *lrf2*, *elf1* were the highest regulon specificity score (RSS) in ECM CAFs and wound healing CAFs respectively. Further, these regulons were all related to immune regulation. We speculated that the two CAFs subtypes played an important role in immune regulation, which might be immunosuppressive [39,66,67] (Fig. 9F). By comparing the cell communication between Control group and PD1+E0771M-AP NPs group, the association between ECM CAFs, wound healing CAFs and tumor vessels (tumor vEC) in PD1+E0771M-AP NPs group was significantly blocked. In contrast, the association between inflammatory vessels and CD8^+^ memory T cells was significantly enhanced in PD1+ E0771M-AP NPs group (Fig. 9G). These results supported the conclusion that ARS increased T cells infiltration in tumor based on the proportion of inflammatory vessels increased. These two subtypes of CAFs were closely associated with tumor proliferation and immunosuppression. The solid tumor loosening agent prepared by us could specifically inhibit ECM CAFs and wound healing CAFs function status and regulate tumor SWE imaging stiffness. Therefore, we provided a theoretical basis for artificial intelligence to SWE images deep learning.

## 3 Discussion and conclusion

The correlation between the tumor imaging features and tumor tissue pathological features has been studied for a long time. Lymph node metastasis of thyroid cancer was predicted using artificial intelligence (AI)-based deep learning of ultrasound images [68]. Each pixel of the ultrasound image was quantified to summarize the imaging features of thyroid cancer with lymph node metastasis. SWE imaging stiffness in TNBC was higher than that in HER2^+^ breast cancer and ER^+^ breast cancer [14]. In addition, the high SWE stiffness region was consistent with the hypoxic region [13], which could help assess the pathological features of patients in a non-invasive way. The association between imaging features and pathological features are well studied; however, the mechanism underlying this association remained unknown. In this study, we demonstrated that SWE imaging stiffness in TNBC was higher than that in non-TNBC, due to the increased fibrosis and more activated fibroblasts in TNBC patients. CAFs are an important component of the solid tumor microenvironment [[2], [3], [4], [5]]; they will lead to a worse TME. This should be considered while studying breast cancer. Based on the pathological data from 233 patients, we found that TNBC patients had a higher Ki67 value and a lower CD8^+^ T cell infiltration rate in the tumor than that in the non-TNBC patients. CAFs are activated in response to tumor injury; these could secrete extracellular matrix and release proteolytic enzymes. CAFs are the main sources of vascular growth factors, hepatocyte growth factors, and stromal cell derived factors, all of which play an important role in tumor proliferation, metastasis, and drug resistance [3,4]. In the clinical context, patients experience varying remission rates with neoadjuvant therapy. Therefore, we proposed a model for predicting neoadjuvant response efficacy through SWE imaging and obtained a good correlation.

SWE stiffness is related to the CAF content in tumors. Fibrosis occurs in tumor tissue during the treatment period [23], and the SWE imaging stiffness decreases with the treatment progress. This cannot completely explain the mechanism of SWE imaging. We selected artesunate, a drug with anti-fibrosis effect, for targeting CAFs; however, it has a short half-life *in vivo*. Nanoparticles can improve the anti-fibrosis effect of ARS by enhancing accumulation in the tumor. However, due to excessive retention in the liver and spleen, their effectiveness is reduced [69]. Therefore, we exploited the homologous aggregation and immune escape properties of tumor cells to confer the nanoparticles with targeting ability and to enhance their circulation *in vivo* [32,70]. In this study, biomimetic nanoparticles homologous aggregated into the tumor and gradually penetrated the tumor stroma after circulating *in vivo* for more than 4 h, which is line with earlier reports [53,58]. However, part of the biomimetic nanoparticles injected intravenously into mice were still metabolized through the kidney.

Hypoxia in tumor is often closely related to immunosuppression. There are several reasons for hypoxia in tumor, such as high oxygen consumption of tumor cells and decreased blood supply [71]. The high CAF functional status will enhance the protective ability on tumor cells; this can weaken the anti-tumor effect of chemotherapy or immunotherapy [5]. Artesunate inhibited the functional status of CAFs, which reduced the secretion of collagen and vimentin. This resulted in a decrease in stiffness of SWE imaging. In addition, the CAF inhibition significantly enhanced the antitumor effect of paclitaxel and PD1 inhibitor. Usually, CAFs are most active at the edge of the tumor [72]. In clinical practice, tumors have stiff rim sign on SWE imaging [73], this undoubtedly proved the correctness of our conclusion (the tumor SWE imaging stiffness was associated with CAFs functional status) from the side. ARS remodeled the TME and the vessels, increasing oxygen transport to the tumor. Single-cell sequencing indicated a lower proportion of ECM CAFs and wound healing CAFs, both of which belong to the high level functional CAFs clusters, in the PD1+E0771M-AP NPs group. In addition, a higher proportion of vascular endothelial cells were detected in this group, and among these endothelial cells, inflammatory EC were increased significantly. The blood vessels comprised by the inflammatory endothelial cells provided more oxygen to the tumor and enhanced the infiltration of T cells in tumor.

**Scheme. 2:**
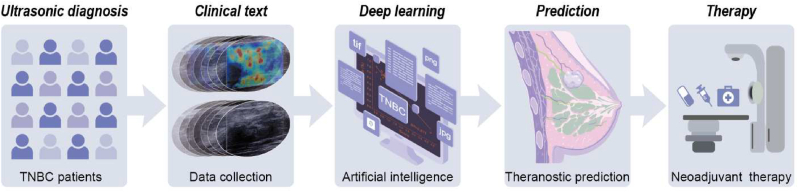
Schematic illustration of our study significance in translation medicine. Our work lays the foundation for the application of artificial intelligence based deep learning shear wave elastic imaging in clinical treatment prediction of TNBC patients.

However, this study has certain limitations; the actual clinical application was not evaluated. The number clinical samples used was small. Therefore, follow-up studies with a larger sample size are warranted to increase the accuracy of these results. In the future, we aim to explore computational algorithms for designing a software tool for the prediction and evaluation of neoadjuvant therapy efficacy in clinical practice [68]. The biomimetic nanoparticle has certain clinical translation significance, as an adjuvant for sensitizing to chemotherapy and immunotherapy (Scheme 2).

## 4 Materials and methods

### 4.1 Materials

PLGA, lactide: glycolide = 50:50, Mw24000-38000 (Sigma-Aldrich), paclitaxel (P1632, Tci), artesunate (88495-63-0, Adamas), field emission transmission electron microscope (Talos F200S, ThermoFisher), particle size distribution analyzer (Malvern), spectrophotometer (nanodrop 2000c, ThermoFisher), enzyme-labeled instrument (synergy H1, Biotek), Fourier infrared spectrometer (Nicolet Avatar 370, Nicolet Avatar), SWE instrument (SuperSonica Aixplorer US scanner, with a 7–15 MHz linear transducer), Bio-Imaging instrument (Smart-LF, Vieworks VISQUE), multimodal imaging instrument (Vevo LAZR, FUJI), flow cytometer (FC500 MPL, Beckman Coulter), confocal laser scanning microscopy (TCS SP5Ⅱ, leica); chemiluminescence meter (Amersham imager 600). MDA-MB-231 (231), MDA-MB-468, MCF-7, SK-BR-3, T-47D, L-929 cells were kindly provided by Cell Bank/Stem Cell Bank, Chinese Academy of Sciences, AU565, E0771, CAFs, cells were provided by Department of Breast Surgery, Shanghai Cancer Center, HMEC, THP-1 cells were provided by Department of Nuclear Medicine, Fudan University Shanghai Cancer Center. Mice were purchased from GemPharmatech. Breast cancer samples in tissue chip were donated by patients, all samples were obtained after informed consent and approval from patients (approved by Fudan University Shanghai Cancer Center Institutional Review Board, SCCIRB, 2107238–18). SWE image processing was performed by Osirix Lite. Single-cell sequencing services were provided by OE Biotech Co., Ltd. (Shanghai, China). The sequencing and bioinformatics analysis were performed by All statistical analyses, unless otherwise specified, were conducted using R. The use of cell lines in our experiments received the approval of the ethical review board of shanghai cancer center, Fudan University. All of the animal experiments were performed according to the protocols approved by the department of laboratory animal science, Fudan University.

## 5 Methods

### 5.1 Preparation of nanoparticles

AP NPs was synthesized by oil-in-water single emulsion solvent volatilization method [74]. Briefly, 10 mg ARS was dissolved in methanol and mixed with dichloromethane (DCM) solution with 100 mg PLGA for emulsification. After emulsification, the suspension was volatilized at room temperature for 5 h, followed by gradient centrifugation, and finally the nanoparticles were obtained.

### 5.2 Preparation of E0771/MDA-MB-231 cell membranes

After the cells covered the dish were rinsed with PBS, the hypoosmolality RIPA lysate containing 1% phenylmethanesulfonyl fluoride (PMSF) was added and lysed at 4 °C for 30min. The supernatant was collected after suspension centrifugation at 12000 rpm for 8 min. Next, the collected supernatant was centrifuged again (20000 rpm, 1 h, 4 °C). Finally, the cell membrane was collected.

### 5.3 Preparation of biomimetic nanoparticles

Biomimetic nanoparticles could be obtained by ultrasonic 15min of suspension of biofilm and nanoparticles.

### 5.4 Characteristic of nanoparticles

To measure the size of nanoparticles in aqueous solution and biomimetic nanoparticles aqueous solution at different times (1, 2, 3, 4, 5, 6 day), the suspension was added into a quartz dish for assessing, and the suspension was blown evenly with dropper before each measurement. The surface potential of nanoparticles and biomimetic nanoparticles was measured by particle size distribution analyzer.

NP formulation powder was prepared by lyophilization method. The FT-IR spectra of ARS, PLGA, and AP NPs were recorded on a FT-IR spectrometer. Background scanning and correction were carried out before each measurement.

### 5.5 Drug standard curve and drug-loading capacity

Artesunate were dissolved in methanol to prepare ARS solutions with different concentrations (ARS: 10, 20, 40, 80, 100, 200, 400, 500, 1000 μg/mL). Pure methanol solution was used for blank baseline scanning, and the absorbance of ARS methanol solutions with different concentrations at 222 nm were measured.

To measure the drug-loading capacity of nanoparticles, a certain amount of nanoparticles were suspended in water, the suspension was heated to 60 °C until the water dried, and then methanol solution was added to dissolve and mix the powder at the bottom of bottle. The supernatant was collected after centrifugation (12000 rpm, 10min). Finally, measured the absorbance of the solution. Drug-loading capacity = weight of drug in NPs/weight of NPs.

### 5.6 Immune escape experiment of biomimetic nanoparticles

THP-1 cells were suspended in culture dishes and Phorbol 12-myristate 13-acetate (PMA) (ab120297) was added to induce macrophages adhere to the wall. 12 h later, 500 μL of biomimetic nanoparticles or nanoparticles coated with Did fluorescent dye (1 mg/mL) were added into dish. At the end of co-incubation, paraformaldehyde was added for fixation. PBS washing three times and immunostaining blocking solution was added for blocking at room temperature for 1 h. Next, DAPI staining solution was added for staining for 10min, and PBS washed three times. Finally, the endocytosis of the THP-1 cells was observed under confocal laser scanning microscope.

### 5.7 Cytotoxicity assay

The breast cancer cells were treated with different concentrations of ARS solution (0, 5, 10, 15, 20, 30 μM) for 24 h, and then the supernatant was discarded and added into CCK-8 for incubation. Similarly, different concentrations of AP NPs solution or 231M-AP NPs (0, 10, 20, 50, 100, 200 μg/mL) were co-incubation with different cell lines for 24 h respectively, and then determined OD value.

### 5.8 Western blot assay

Cell sample: The cells were seeded into 6-well plate. The control group and 231M-AP NPs group were cultured in normal medium, and the other groups were cultured in 231 or E0771 conditioned medium (the medium of culturing tumor cells for 72 h). In addition, the medium is changed every 3 days medium in 231M-AP NPs groups were also changed every three days with new medium containing 231M-AP NPs. After 216 h, cell proteins were extracted.

Tissue sample: the tumor tissue was mixed with NP-40, and two steel balls were added. After grinding by tissue grinding machine, supernatant was taken after centrifugation, and BCA quantification was performed.

The processed proteins were successively added to the gel tank and electrophoresis solution was added for electrophoresis. After electrophoresis, PVDF membrane was used for membrane transfer. After blocking, the membrane treated with primary antibody were incubated at 4 °C overnight, and then washed with TBST. HRP-Conjugated Second Antibody (SA00001-2, Proteintech) was incubated with membrane at room temperature for 1 h.

Primary antibodies include CD47 Antibody, Galectin-3 Antibody (AF-4670-SP, AF-1154-SP, R&D Systems), Galectin-1 Antibody (11858-1-AP, Proteintech), MMP-14 antibody (29111-1-AP，Proteintech), Anti-TGF beta1 antibody (ab215715，Abcam), α-SMA antibody (14395-1-AP, Proteintech; AF1507, Beyotime; MAB1420-SP, R&D Systems), Phospho-Smad3 (Ser423/425) antibody (#9520, CST), DDR1 antibody (#5583, CST), HIF-1α antibody (#36169, CST), GAPDH (#5174, CST).

### 5.9 Cellular immunofluorescence

The cells were seeded in confocal dishes at low concentrations. The control group and 231M-AP NPs group were cultured in normal medium, and the other groups were cultured in 231 or E0771 conditioned medium (the medium of culturing tumor cells for 72 h). After 72 h incubation in incubator, nanoparticles were added into the corresponding groups and incubated for another 72 h. Next, discard the old medium and washed by PBS. Then, fixation by paraformaldehyde for 15min at room temperature (RT). After PBS Washed for three times, blocked at RT for 1 h. The primary antibody (vimentin antibody, 10366-1-AP; ACTA2 antibody, 14395-1-AP, Proteintech) was incubated with cells at 4 °C overnight. Next, TBST washed for three times. The second antibody (FITC-goat anti rabbit IgG antibody, SA00003-2) was incubated with cells at RT for 1 h. After TBST washed for three times, and PBS added into dish, the dishes were observed under CLSM.

### 5.10 Homologous aggregation targeting *in vitro*

The nanoparticles coated with Did fluorescent dye were divided into three groups: NPs, 231M-NPs, and E0771M-NPs. Cells were treated with different ways to observe the targeting of nanoparticles. During the incubation process, the dish was slightly shaken by a shaker.

### 5.11 Establishment of mice carcinoma in situ model

The 231 cells were suspended in PBS at a density of 1.5 × 10^6^, the matrix gel and cell suspension were mixed evenly at a ratio of 1:1. The 231 cells were planted in the third pair of mammary pads of 4-week-old female nude mice. Animal experiments were carried out five days later.

The E0771 cells were suspended in PBS at a density of 8 × 10^5^ and planted in the third pair of mammary pad of 4-week-old female C57/B6J mice. Animal experiments were carried out seven days later. Tumor volume = length × width^2^/2.

### 5.12 Anti-tumor experiment of biomimetic nanoparticles *in vivo*

Nude mice were randomly divided into four groups: control, ARS, AP NPs, and 231M-AP NPs. The drug was injected via tail vein every three days (dose: ARS 40 mg/kg; NPs 30 mg/mL, 200 μL). Tumor and body weight were measured every three days.

### 5.13 SWE imaging of tumor

SWE imaging was performed on the mouse tumor three days before the end of the treatment cycle. Region of interest (ROI) with a diameter of 1 mm was selected from the hardest three sections to measure the stiffness value, and calculate the average value. The stiffness value of normal tissue at the same depth of tumor was measured by same size ROI to exclude the interference caused by the depth of tumor, but this data was not used for analysis. The mice SWE imaging performer was a professional sonographer with more than 5 years of clinical experience.

### 5.14 In *vivo* experiment of 231M-APP NPs sensitizing chemotherapy

Mice were randomly divided into four groups: control, PTX, PTX + ARS, 231M-APP NPs. Drugs were injected via tail vein every three days (dose: ARS 30 mg/kg; PTX 12 mg/kg; NPs 20 mg/mL, 200 μL). Tumor and body weight of mice were measured every 3 days.

### 5.15 *In vivo* experiment of E0771M-AP NPs sensitizing immunotherapy

Mice were randomly divided into six groups: control, ARS, PD1, ARS + PD1, E0771-AP NPs; PD1+E0771M-AP NPs. Drugs were injected via tail vein every three days (dose: ARS 30 mg/kg; NPs 20 mg/mL, 200 μL). PD1 inhibitor was injected intraperitoneally (dose: 10 mg/kg/twice a week); Tumor and body weight of mice were measured every 3 days.

### 5.16 Photoacoustic evaluation of blood oxygen level in solid tumor

To assess the TME of solid tumor after E0771M-AP NPs (soil loosening agent) remodel, five groups (control, ARS, ARS + PD1, E0771M-AP NPs, PD1+E0771M-AP NPs) were evaluated at day 15 of treatment. Five mice were selected from each group, and tumors were evaluated by Vevo LZAR multimodal imaging instrument. Probe frequency is 21 MHz.

### 5.17 Flow cytometry evaluated the immune response *in vivo*

The blood was collected into anticoagulant tube containing heparin, and then the blood cell lysate was added. After lysis at room temperature for 15min, centrifugation at 1500 rpm for 10min. Repeat these steps until red blood cells were lysis completely. The cells were suspended in the PBS buffer and incubated with antibodies (FITC-65077, Proteintech; 100515, 100707, Biolegend) at 4 °C for 30min. PBS buffer washed three times, and the cells were suspended in 500 μL PBS buffer to evaluate through Flow cytometry.

Mouse spleens were taken, crushed, and suspended in PBS buffer. The cell suspension was filtered with 70 μm-strainer, and the suspension was filtered again with 40 μm-strainer. Added RBC lysate until RBCs were lysis completely. The cells suspension was incubated with antibody (FITC:CD3^+^; PE:CD8^+^; APC:CD4^+^) at 4 °C for 30min. PBS buffer washed three times, and the cells were suspended in 500 μL PBS buffer to evaluate through Flow cytometry.

### 5.18 Correlation analysis between SWE stiffness and tumor pathological indexes

The SWE imaging data and pathological data of 131 patients clinically diagnosed as TNBC and 102 patients clinically diagnosed as non-TNBC were extracted from our department database. Pathology reports (reports come from before any drug treatment) were independently diagnosed by two pathologists. The stiffness value of tumor was obtained by measuring the ROI (diameter 4 mm) of the hardest part of the tumor. At the same time, the patient's pathological indicators were quantitatively scored.

### 5.19 Tissue chip quantitative evaluation

Immunohistochemical results of tissue chip were quantified by the Pharmaservice provide by Indica Labs.

### 5.20 Correlation analysis between stiffness and neoadjuvant efficacy

Fourteen patients diagnosed with TNBC were selected from the database. The reduction of tumor volume (i.e., remission rate) was calculated according to the tumor size measured by ultrasound. The period was from the time of the first ultrasound diagnosis to the end of the time of the last ultrasound diagnosis. The stiffness value (ROI diameter 4 mm) was measured at the hardest part of the maximum section of the tumor by SWE image. The long diameter (a) and short diameter (b) at the maximum section were measured by the RadiAnt Dicom Viewer.

### 5.21 Behavioral analysis of E0771M-NPs agent in mice

Cypate is encapsulated in biomimetic nanoparticles. The NPs suspension (4 mg/mL, 200 μL) was injected into the body via tail vein. The distribution of nanoparticles in mice was observed at different time points (0, 0.5, 1, 2, 4 h). The signal intensity of tumor was quantified.

Six hours after the tail vein injection, the mice organs were taken for imaging to observe the distribution of nanoparticles in each organ.

Did-loaded biomimetic nanoparticles suspension (4 mg/mL, 200 μl) was injected into body via vein, and the tumor tissue was collected at 1 h and 4 h after injection. The experiment was repeat twice.

### 5.22 Preparation of single-cell suspensions

The fresh tissue samples were lysed in 1 mL nuclear lysate for 7min. Further, 1 mL wash buffer was added into the suspension and filtered by 40-μm cell strainers, centrifuged the filtered suspension (500 g, 5min, 4 °C). The nuclei were diluted with PBS+1% BSA to 700–1200/μL. Follow the instruction of 10 × Genomics Chromium Next GEM Single Cell 3ʹ Reagent Kits v3.1（1000268）and cDNA library amplification. Chromium™ Single cell 3'/5′ Library Construction Kit (1000020) was used for DNA library construction. Finally, the library was sequenced on Illumina Nova 6000 platform using PE150 sequencing mode.

### 5.23 Dimension reduction and cluster analysis

The FindVariableGenes function in Seurat package was used to screen highly variable genes (HVGs). Principal component analysis (PCA) was performed using the expression profiles of HVGs, and the results were projected in two-dimensional by UMAP.

### 5.24 Marker gene identification

The FindAllMarkers function in Seurat package was used for marker gene identification, which is to find genes who are differentially up-regulated in each cell classification compared with other cells. VlnPlot and FeaturePlot were used to visualize the identified marker genes.

### 5.25 Cell type identification

The singleR package was used to calculate the correlation between the expression spectrum of the cells to be identified and the reference data set, and then the cell types with the highest correlation in the reference data set were assigned to the identified the cells.

### 5.26 Gene pathway enrichment analysis

We performed differential gene analysis for PD1 group and PD1+E0771M-AP NPs group based on FindMarkers function. Finally, differential genes were analyzed by GO enrichment. Differential gene screening criteria: p < 0.05, fold change: 1.5.

### 5.27 GSEA analysis

Gene Set Enrichment Analysis (GSEA) was performed on the all genes detected in group PD1+E0771M-AP NPs and group PD1 to compare the total number of genes under specific gene sets. The analysis method was *signal to noise*.

### 5.28 Clinical samples

From January 2019 to December 2021, we recruited 233 breast cancer patients and divide them into TNBC group (n = 131) and non-TNBC (n = 102). The SWE imaging images and pathological data were collected. Data were collected from patients before treatment or only after surgical resection.

From January 2013 to December 2019, we recruited 100 breast cancer patients who only achieved surgery. The breast cancer tissues were collected.

From January 2020 to December 2021, we recruited 14 breast cancer patients who had achieve neoadjuvant therapy. They were followed up until surgical resection. If the patient's condition deteriorates after two courses of neoadjuvant therapy, the neoadjuvant therapy will be regarded as failure, and the follow-up will end. The SWE imaging images and pathological data were collected.

### 5.29 Statistical analysis

The data were collected from at least two independent experiments and all data in different experimental groups were used mean ± SD to express. Differences between groups were tested with *t*-test or one-way ANOVA. The significance of differences is indicated at *p < 0.05, **p < 0.01, ***p < 0.001, and ****p < 0.0001.

## Credit author statement

Conceptualization: Dongdong Zheng, Hongbo Zhang, Shichong Zhou, Methodology: Dongdong Zheng, Hongbo Zhang, Shichong Zhou, Investigation: Dongdong Zheng, Jin Zhou, Lang Qian, Xuejiao Liu, Visualization: Dongdong Zheng, Hongbo Zhang, Shichong Zhou, SWE imaging: Jin Zhou, Dongdong Zheng, Clinical data collecting: Dongdong Zheng, Lang Qian, Funding acquisition: Hongbo Zhang, Shichong Zhou, Cai Chang, Supervision: Cai Chang, Shuang Tang, Hongbo Zhang, Shichong Zhou, Writing – original draft: Dongdong Zheng, Shichong Zhou, Writing – review & editing: Dongdong Zheng, Hongbo Zhang, Shichong Zhou.

## Ethics approval statement

Breast cancer samples in tissue chip were donated by patients, all samples were obtained after informed consent and approval from patients (approved by Fudan University Shanghai Cancer Center Institutional Review Board, SCCIRB, 2107238–18). All of the animal experiments were performed according to the protocols approved by the department of laboratory animal science, Fudan University.

## Funding

National Natural Science Foundation of China (NO. 81830058, 82071945, 81401422, 81871472); Shanghai Committee of Science and Technology, China (No. 21S31905400); Research Fellow (Grant No. 328933), project (347897); Solutions for Health Profile (336355), and InFLAMES Flagship (337531) projects from 10.13039/501100002341Academy of Finland, Finland China Food and Health International Pilot Project funded by the Finnish Ministry of Education and Culture.

## Data and materials availability

All data needed to evaluate the conclusions in the paper are present in the paper and/or the Supplementary Materials.

## Declaration of competing interest

The authors declare that they have no competing interests.
