# Supplementary material for: Biomimetic nanoparticles drive the mechanism understanding of shear-wave elasticity stiffness in triple negative breast cancers to predict clinical treatment
Source: Bioact Mater. 2022 Nov 3;22:567–87. doi: 10.1016/j.bioactmat.2022.10.025 (PMC9638718; doi:10.1016/j.bioactmat.2022.10.025)
Supplement: Multimedia component 1 [file mmc1.pdf]

Biomimetic Nanoparticles Drive the Mechanism Understanding of Shear-Wave Elasticity Stiffness in Triple Negative Breast Cancers to Predict Clinical Treatment

Dongdong Zheng[1#], Jin Zhou[1#], Lang Qian[1], XueJiao Liu[4], Cai Chang[1], Shuang Tang[3, 5*], Hongbo Zhang[2, 6, 7*], Shichong Zhou[1*]

[1]Department of Ultrasound, Fudan University Shanghai Cancer Center, Shanghai 200032, P. R. China;

[2]Pharmaceutical Sciences Laboratory, Åbo Akademi University, FI-00520 Turku, Finland;

[3]Cancer Institute, Fudan University Shanghai Cancer Center, Shanghai 200032, PR China;

[4]Institutes of Biomedical Sciences and Department of Chemistry, Fudan University, Shanghai 200032, P. R. China;

[5]Department of Nuclear Medicine, Fudan University Shanghai Cancer Center, Shanghai 200032, PR China;

[6]Turku Bioscience Center, University of Turku and Åbo Akademi University, FI-00520 Turku, Finland.

[7]The First Affiliated Hospital of Wenzhou Medical University, Wenzhou 325000, P. R. China.

[#]These authors contributed equally to this work

[*]Corresponding author. Email: tangshuang@fudan.edu.cn, hongbo.zhang@abo.fi, sczhou@hotmail.com

Include:
Supplementary methods: 1.1-1.10
Supplementary figures: Fig.S1-Fig.S36

Supplementary methods

1.1. Preparation of Did-loaded nanoparticles

500μL Did (abs47048165, Absin) ethanol solution (1mM) was mixed with DCM solution with 100mg PLGA for emulsification. After emulsification, the suspension was volatilized at room temperature for 5h, followed by gradient centrifugation, and finally the nanoparticles were obtained.

1.2. Preparation of cypate-loaded nanoparticles

Briefly, 10mg cypate (95837-47-1, BioChemPartner) was dissolved in methanol and mixed with DCM solution with 100mg PLGA for emulsification. After emulsification, the suspension was volatilized at room temperature for 5h, followed by gradient centrifugation, and finally the nanoparticles were obtained.

1.3. Preparation of APP NPs

5mg ARS and 5mg PTX was dissolved in methanol and mixed with DCM solution with 100mg PLGA for emulsification. After emulsification, the suspension was volatilized at room temperature for 5h, followed by gradient centrifugation, and finally the nanoparticles were obtained.

1.4. Characteristic of nanoparticles

Powder form of the NP formulation was prepared by lyophilization method. The FT-IR spectra of ARS, PLGA, PTX, APP NPs were recorded on a FT-IR spectrometer. Background scanning and correction were carried out before each measurement. A manual press was used to form the pellets.

Paclitaxel were dissolved in methanol to prepare PTX solutions with different concentrations (PTX: 50, 100, 200, 250, 500, 1000μg/mL). Pure methanol solution was used for blank baseline scanning, and the absorbance of PTX methanol solutions with different concentrations at 273nm were measured.

Artesunate and paclitaxel were dissolved in methanol solution in the ratio of 1:1, and the mixed solutions were measured by spectrophotometer. Finally, based on the above results, the concentration-absorbance standard curve was fitted.

After solvent volatilization, the nanoparticles with different particle sizes were obtained by gradient centrifugation (2000rpm,5min; 8000rpm, 5min; 16000rpm,5min).

1.5. Artesunate combined with paclitaxel sensitization therapy in vitro

MDA-MB-231 cells in logarithmic growth phase were seeded into 96-well plates and adhered for 12 hours. The cells were treated with different ways according to the groups (Control, ARS, PTX, and ARS+PTX; ARS: 20μM, PTX: 20μM). After 24 hours incubation, cell viability was assessed.

MDA-MB-231 cells in logarithmic growth phase were seeded into 96-well plates and adhered for 12 hours. The cells were treated with different ways according to the groups (Control, 231M-AP NPs, 231M-APP NPs, 200μg/mL). After 24 hours, 48 hours, 72 hours incubation respectively, cell viability was assessed.

1.6. Homologous aggregation targeting detected by CLSM in vitro

To enhance the evidence of homologous aggregation targeting effect of tumor cell

membranes, T-47D cells were incubated with NPs, 231M-NPs, and E0771M-NPs respectively. Finally, we observed homologous aggregation around cells.

1.7. Homologous aggregation targeting detected by flow cytometry in vitro

The tumor cells were seeded into 6 well plate. After 12 hours, the groups were divided into MDA-MB-231 group, NPs group (1h, 4h), and 231M-NPs group (1h, 4h) according to different treatment methods. Add nanoparticles and co-culture in incubator, and then free nanoparticles were washed by PBS. Finally, the cells were detected.

1.8. Image J quantized fluorescent section

Adjust the image format to 8-bit format, and image threshold, finally calculate the image optical intensity. The quantization result is the quantization result of the region randomly selected form slice by CLSM.

1.9. Chemotherapeutic sensitization mediated by 231M-APP NPs

MDA-MB-231 cells were inoculated on 96-well plates for 24h, and then cell experiment was performed. The experimental groups were divided into: Control, PTX, ARS, and PTX+ARS. MDA-MB-231 cells were treated with different ways for 24h according to the group. The cell viability was measured by CCK-8 kit. The drugs were suspended in complete medium.

To observe anti-tumor effect of 231M-APP NPs, MDA-MB-231 cells were incubated with 231M-APP NPs and 231M-AP NPs respectively for 24h, 48h and 72h, respectively. Finally, the cell viability of different groups was measured. The drugs were suspended in complete medium.

1.10. RNA velocity analysis

Based on the output results of the Cell Ranger, we used Python scripts velocy.py[1] respectively to calculate RNA velocity values for each gene in each cell and the embedding RNA velocity vector to low-dimension space were performed with the R package velocyto.R v0.6. Velocity fields were projected onto the UMAP embedding obtained in Seurat[2].

Supplementary figures

1.

Ki67	Score
5 （< 10）	0
10	1
15	1.5
20	2
30	3
40	4
50	5
60	6
70	7
80	8
90	9

CD8	Score
5 （< 10）	9
10	8
15	7.5
20	7
30	6
40	5
50	4
60	3
70	2
80	1
90	0

Fig.S1. The list of scores corresponding to different degrees of pathological indicators.

2.

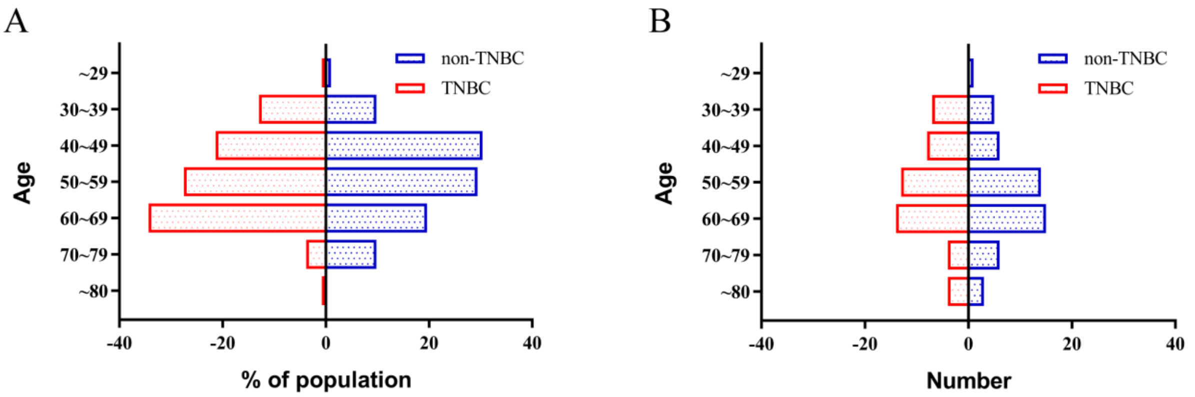

Fig. S2. (A) The pyramid figure of age distribution proportion in TNBC group and non-TNBC group; **(B)** The pyramid figure of age distribution in TNBC group (n=50) and non-TNBC group (n=50) from tissue microarray.

3.

$$Remission\ rate = \frac{(\mathrm{a} \times \mathrm{b})\text{before treatment}}{(\mathrm{a} \times \mathrm{b})\text{after treatment}}$$

Fig.S3. The calculation formula of remission rate (a is length, b is width).

4.

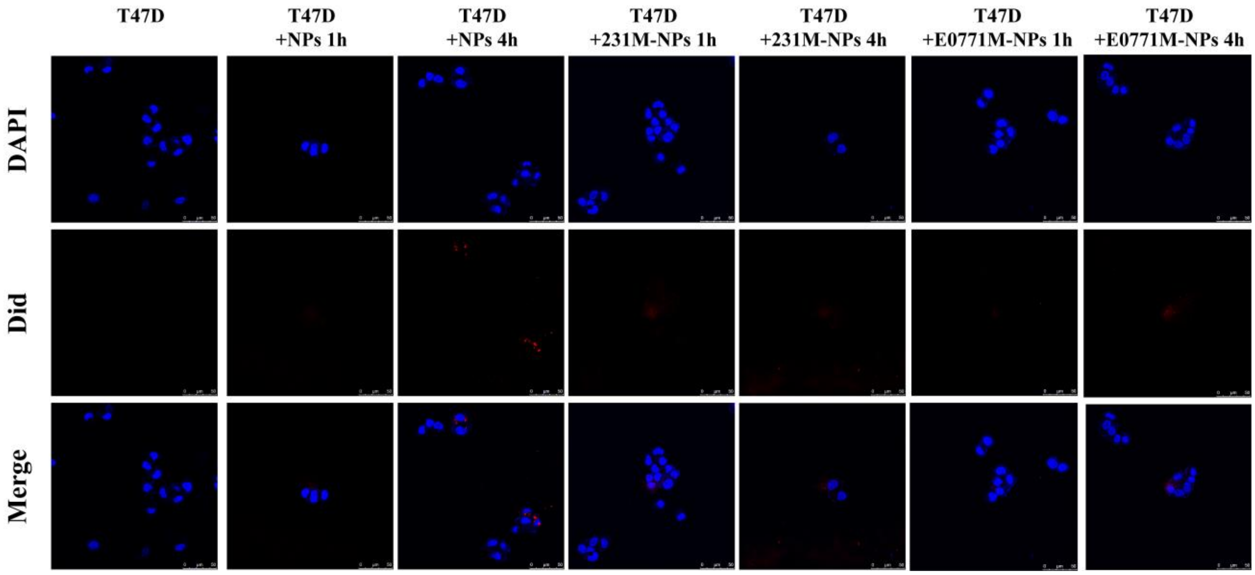

Fig.S4. Homologous targeting of heterotypic cells in vitro: the nanoparticles were labeled with Did, and nanoparticles (red), 231M-NPs (red), and E0771M-NPs (red) were incubated with T-47D cells (blue) respectively, and intracellular fluorescence was observed after co-incubation for 1h and 4h. Scale bar: 50μm.

5.

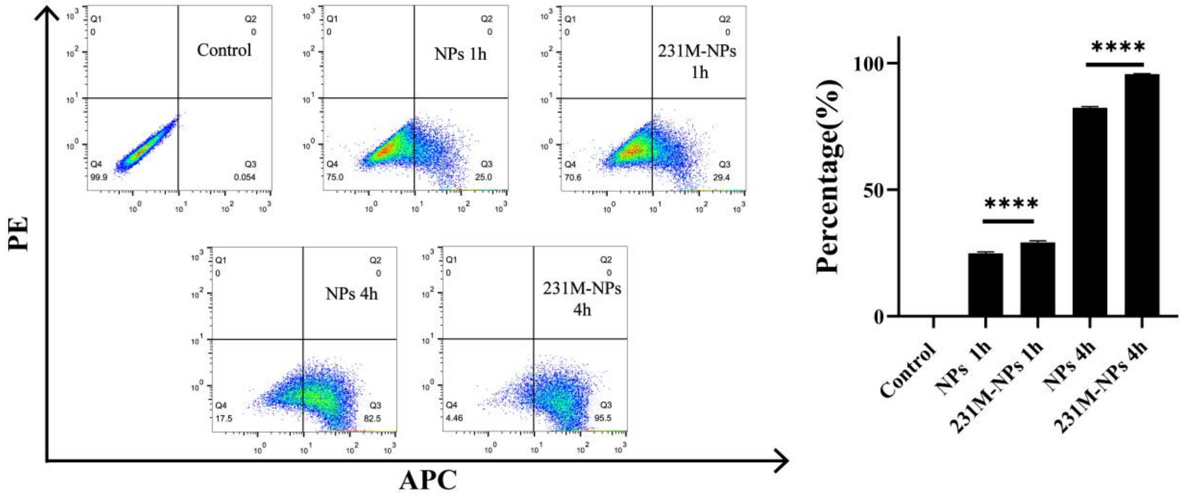

Fig. S5. Flow cytometry analysis and quantification results of homologous aggregation targeting experiment of 231M-NPs in vitro (n=3). Data expressed as mean ± SD.

6.

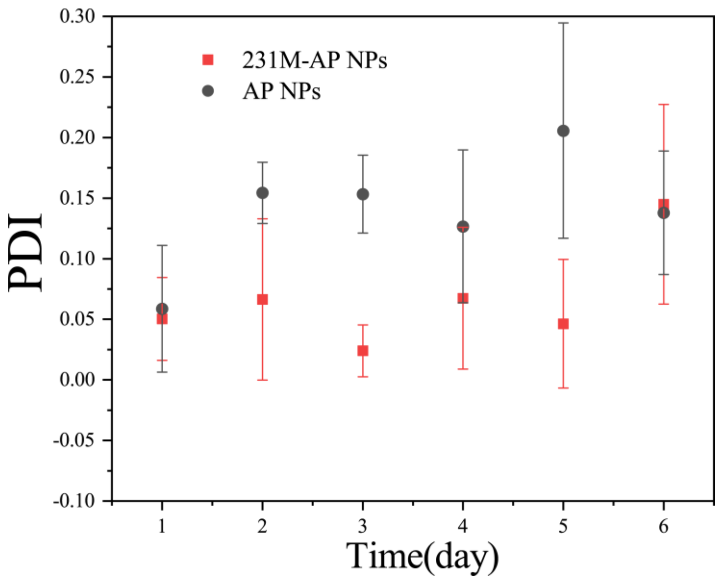

Fig. S6. Polydispersity index (PDI) variations of 231M-AP NPs and AP NPs during 6-day monitoring (n=3). Data expressed as mean ± SD.

7.

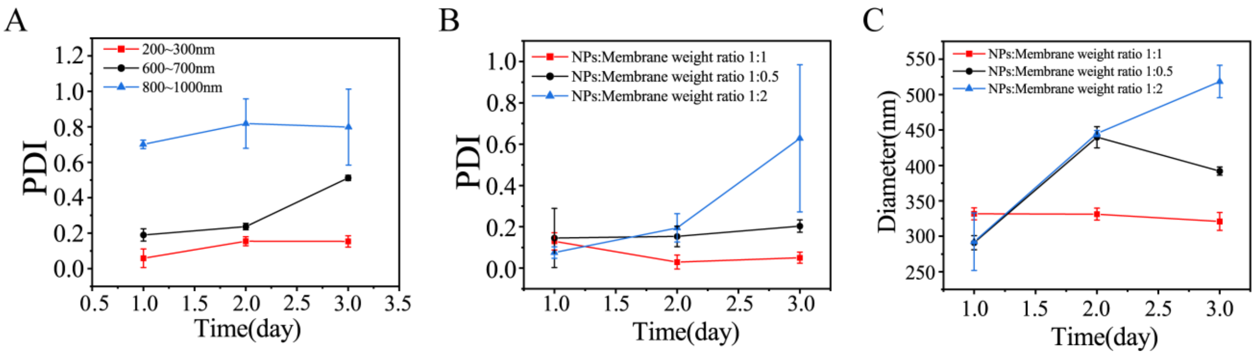

Fig. S7. (A) The PDI-Time curves of nanoparticles with different hydrate particle sizes were obtained from each gradient centrifugation during the nanoparticles preparation (n=3); **(B)** The PDI-Time curves of biomimetic nanoparticles with different nanoparticles: membrane weight ratio (n=3); **(C)** The diameter-time curves of biomimetic nanoparticles with different nanoparticles: membrane weight ratio (n=3).

8.

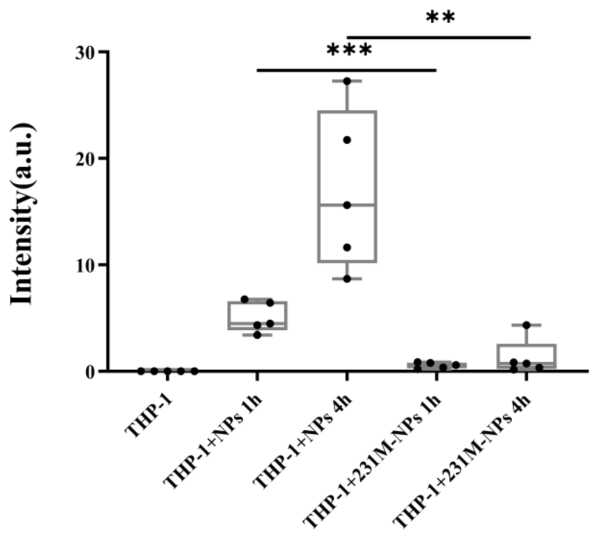

Fig. S8. The quantification result of fluorescence intensity in THP-1 cells to quantify the number of 231M-NPs or NPs phagocytosed by THP-1 cells, n=5, data expressed as mean±SD. **p<0.01, ***p<0.001

9.

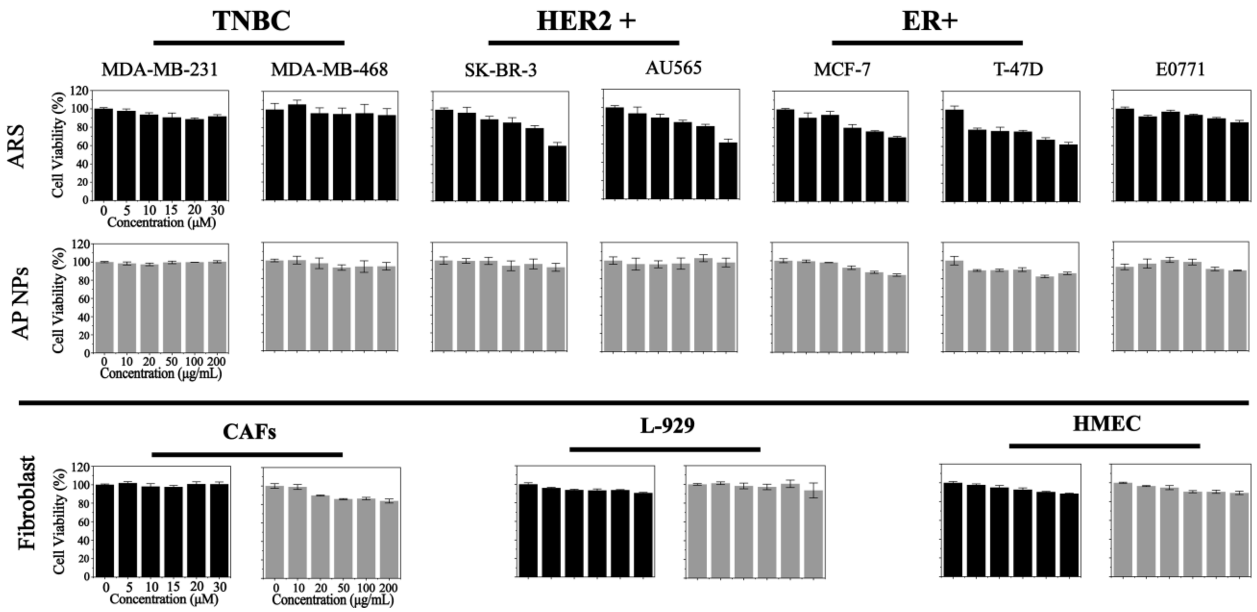

Fig. S9. Cell viability of different cell lines incubated with ARS and AP NPs respectively at different concentrations (the concentration gradient remained consistent in all cell lines) for 24h (n=4). Data expressed as mean ± SD.

10.

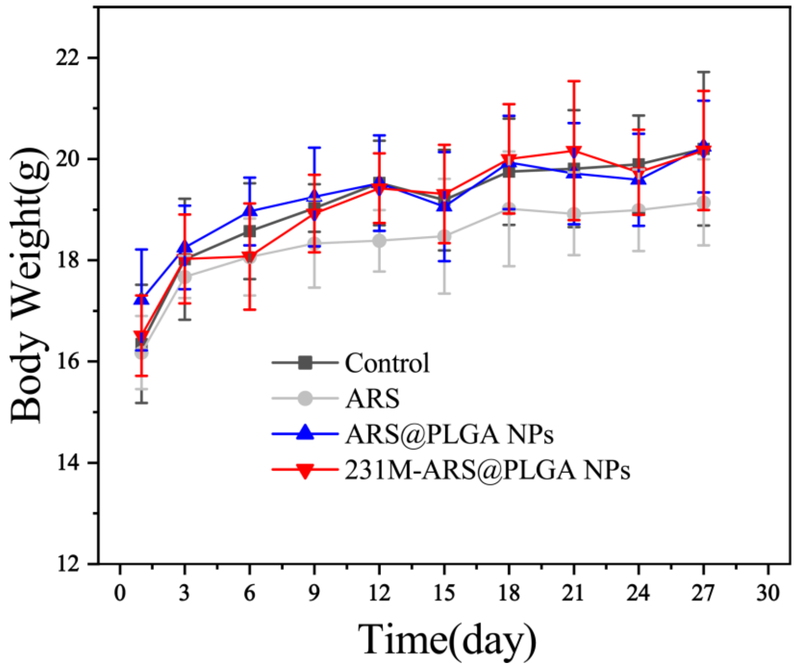

Fig.S10. Body weight monitoring curves of different groups of mice during the 27-day treatment period (n=10). Data expressed as mean ± SD.

11.

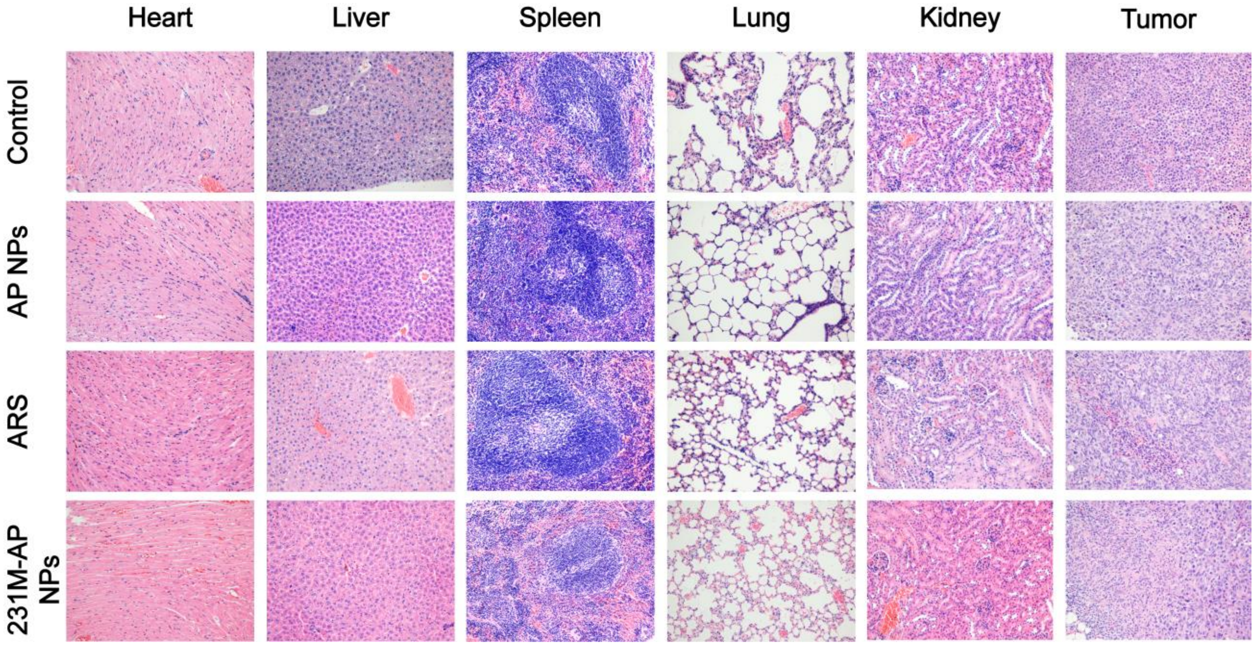

Fig.S11. Microscope images of H&E stained tissues in various organs from different groups (×400 magnification).

12.

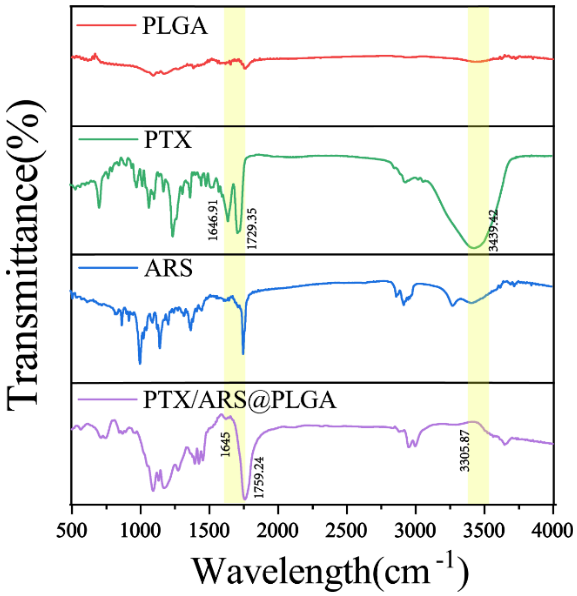

Fig.S12. Fourier infrared spectra of ARS, PTX, PLGA, AP NPs and APP NPs.

13.

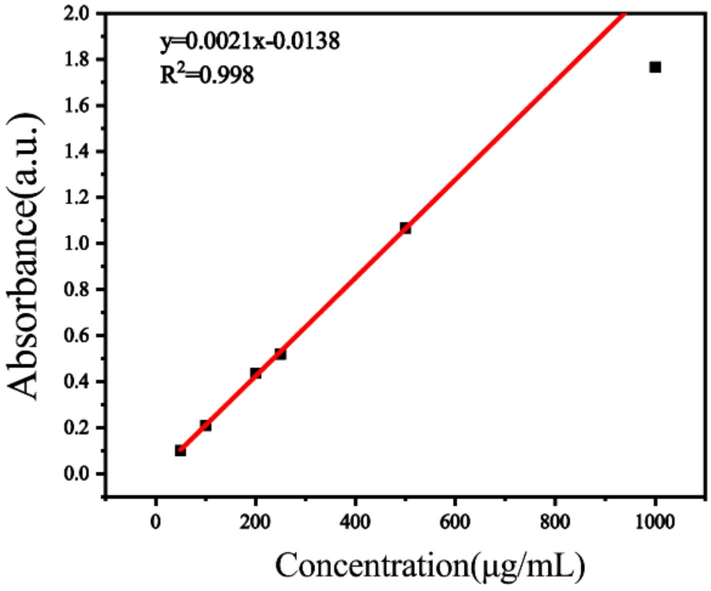

Fig.S13. The concentration-absorbance standard curve of PTX in methanol solution at 273nm (n=3). Data expressed as mean ± SD.

14.

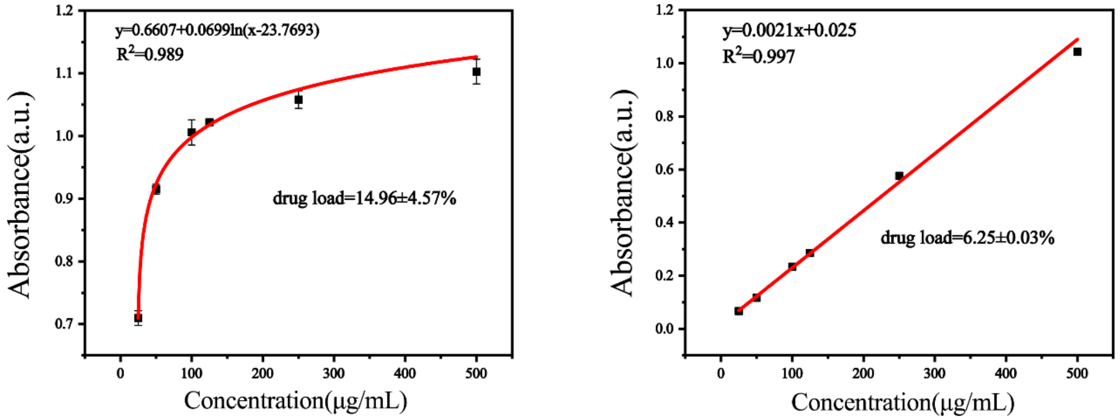

Fig.S14. (A) Concentration-absorbance standard curve of ARS at 222nm in methanol solution mixed with ARS and PTX 1:1; **(B)** Concentration-absorbance standard curve of PTX at 273nm in methanol solution mixed with ARS and PTX 1:1 (n=3). Data expressed as mean ± SD.

15.

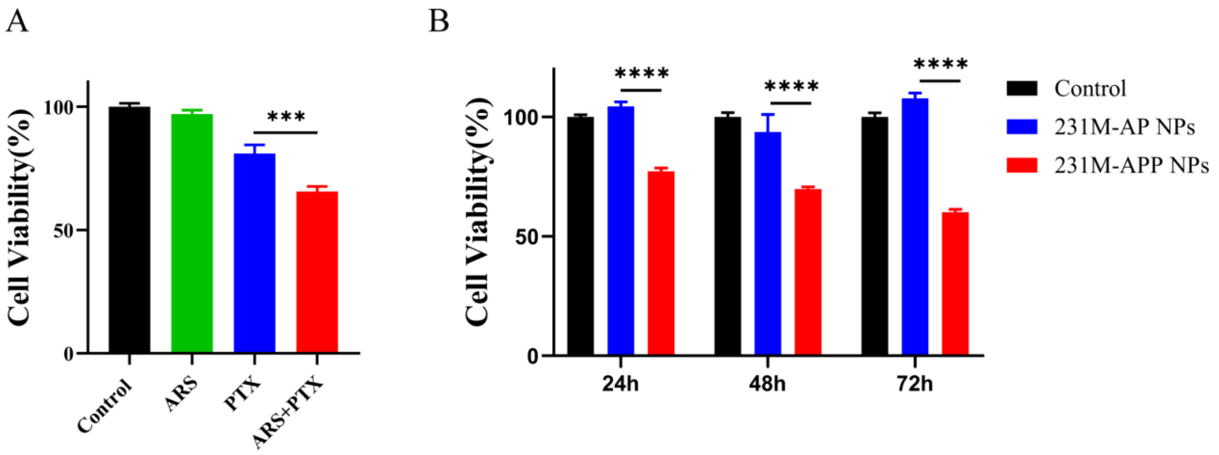

Fig.S15. (A) Cytotoxicity assay of the antitumor effect of paclitaxel (20μM) sensitized by artesunate (20μM) at 24 hours incubation time. **(B)** Cytotoxicity assay of MDA-MB-231 cells incubated with 231M-AP NPs and 231M-APP NPs respectively for different times (24h, 48h, 72h). n=4, data expressed as mean ± SD.

16.

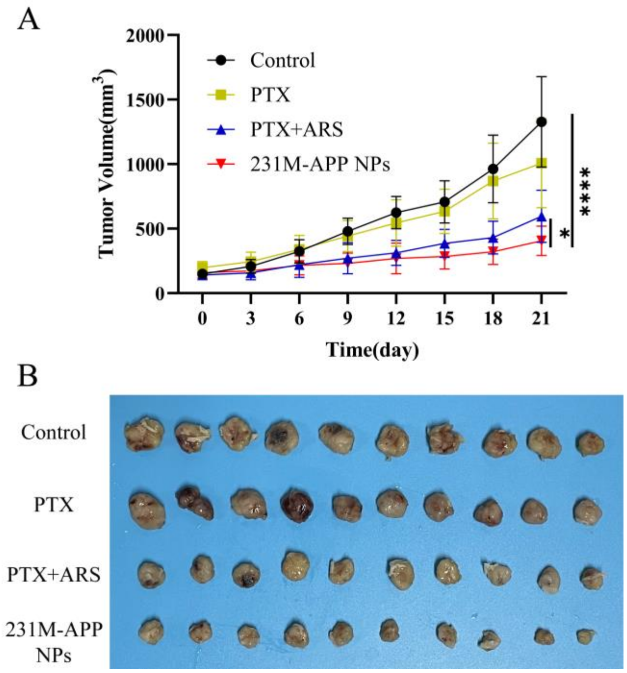

Fig.S16. (A) The curve of tumor volume variations in different groups during the 21-day monitoring period (n=10). **(B)** Photograph of each tumor in different groups of mice on 21st day during the treatment period. Data expressed as mean ± SD.

17.

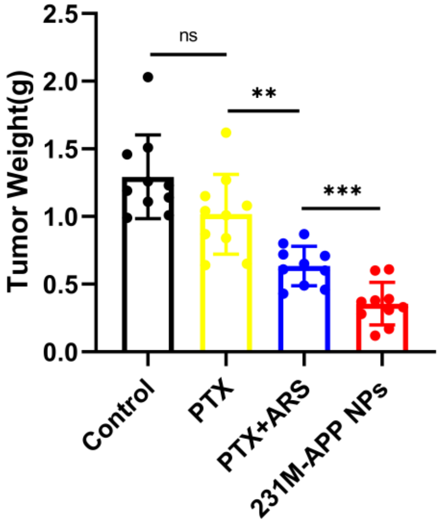

Fig.S17. Tumor weight of mice in different groups (n=10). Data expressed as mean ± SD

18.

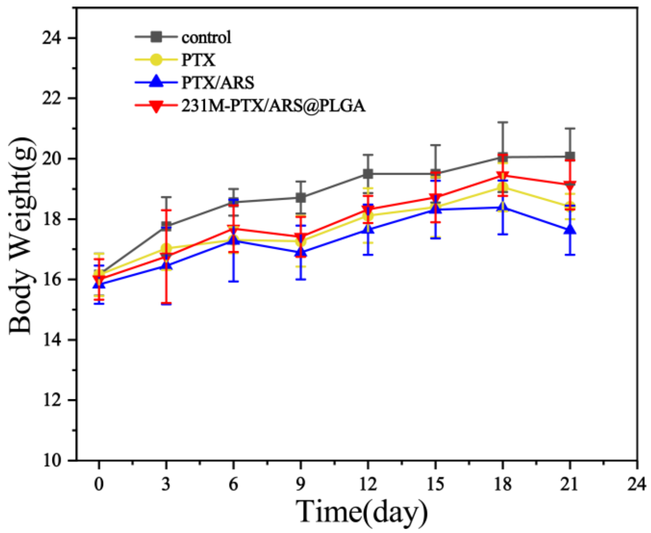

Fig.S18. Body weight monitoring curves of different groups of mice during the 21-day treatment period (n=10). Data expressed as mean ± SD.

19.

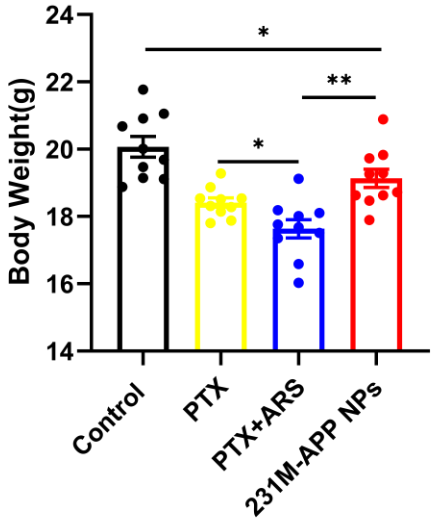

Fig.S19. Body weight of mice in different groups on the last day of treatment (n=10). Data expressed as mean ± SD.

20.

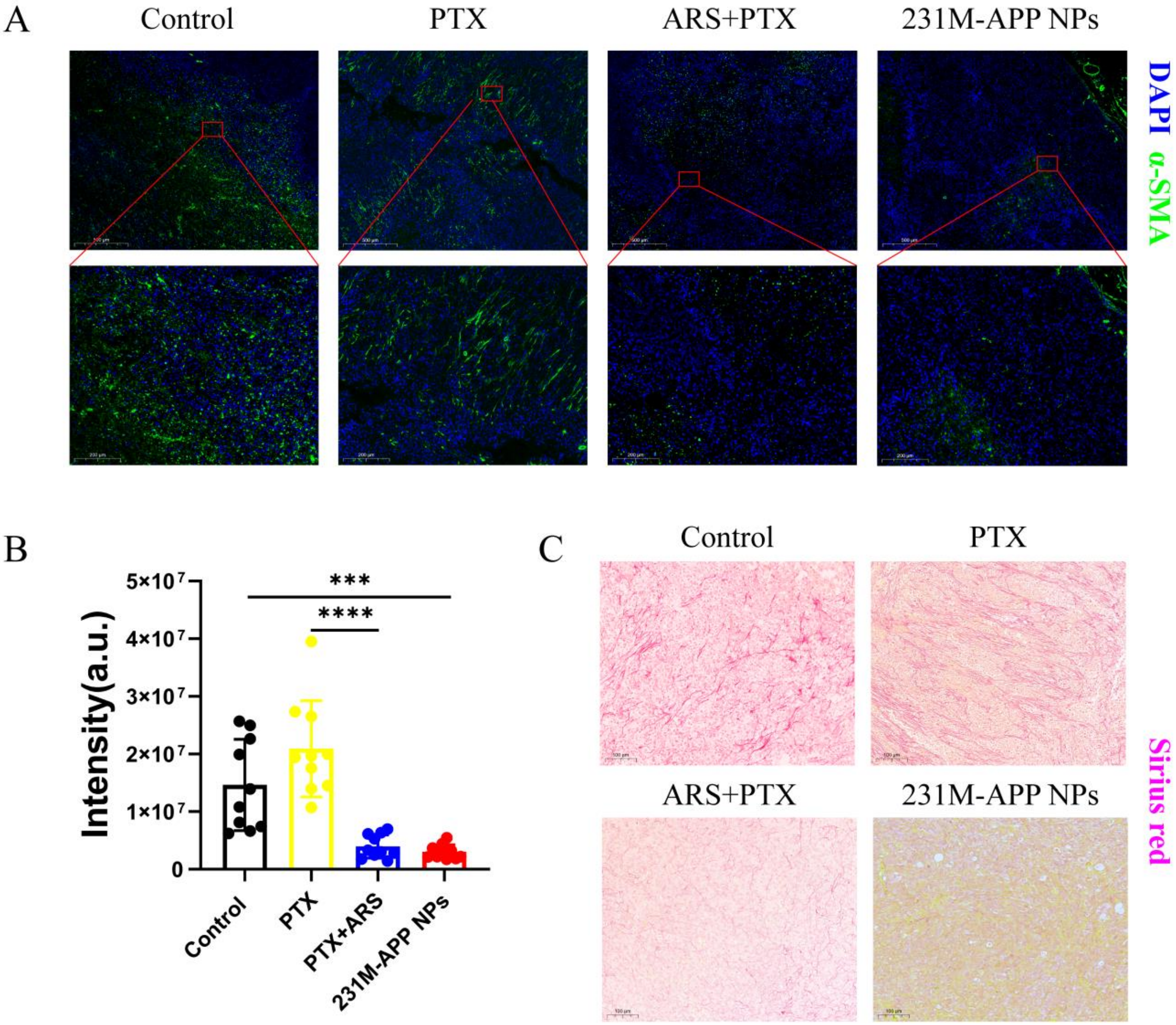

Fig. S20. (A) Representative immunofluorescence images of α-SMA (green) staining of tumor tissue (blue) in different groups. Scale bar in low magnification: 500μm, scale bar in high magnification: 200μm; **(B)** Quantification of α-SMA biomarkers in tissue immunofluorescence sections (n=10). Data expressed as mean ± SD. **(C)** The Sirius red staining images of tumor tissue in different groups, scale bar=100μm.

21.

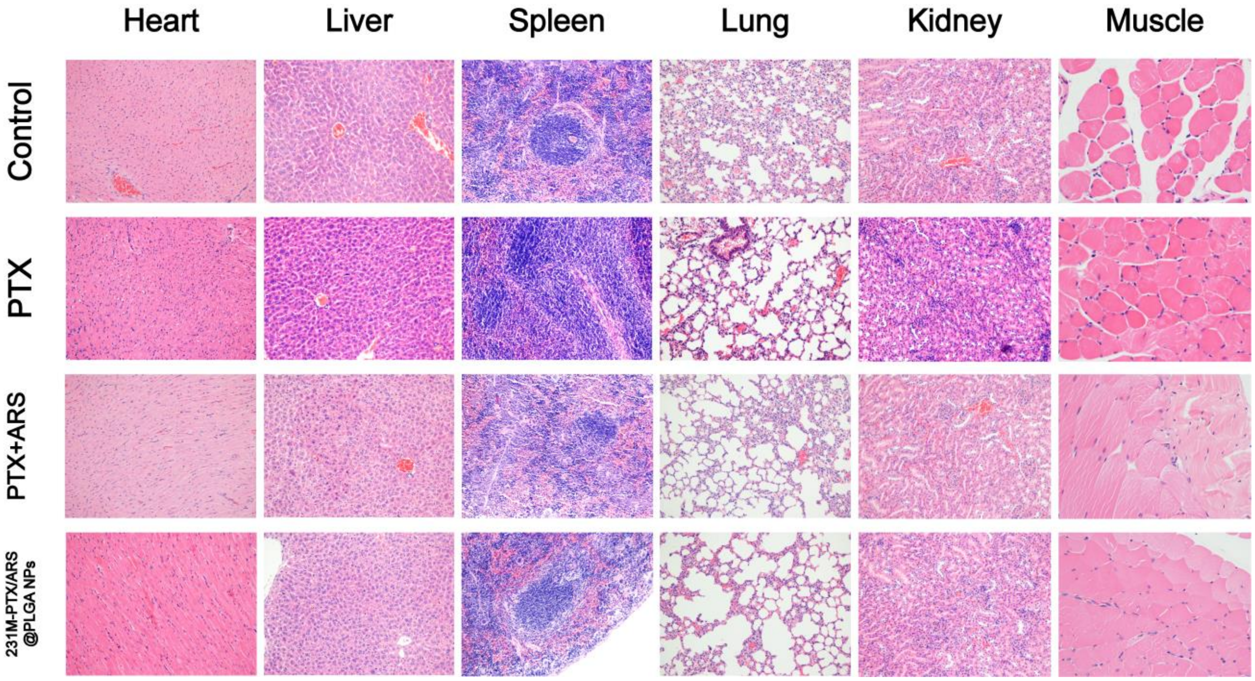

Fig.S21. Microscope images of H&E stained tissues in various organs (×400, magnification).

22.

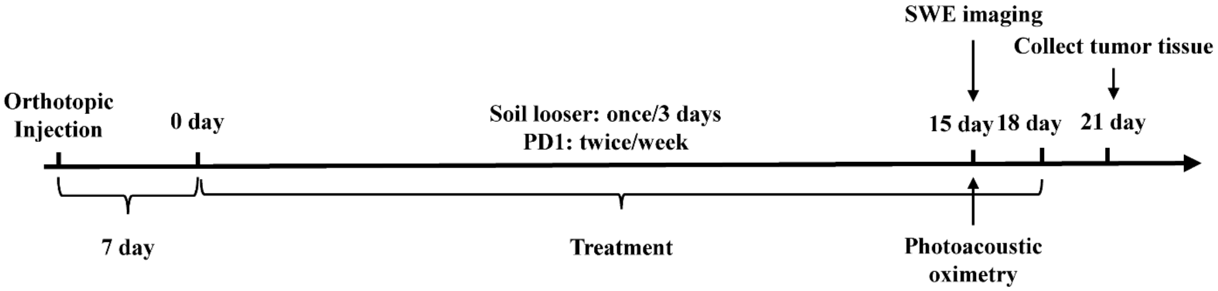

Fig. S22. Diagram shows the experimental course of orthotopic mice modal (n=10).

23.

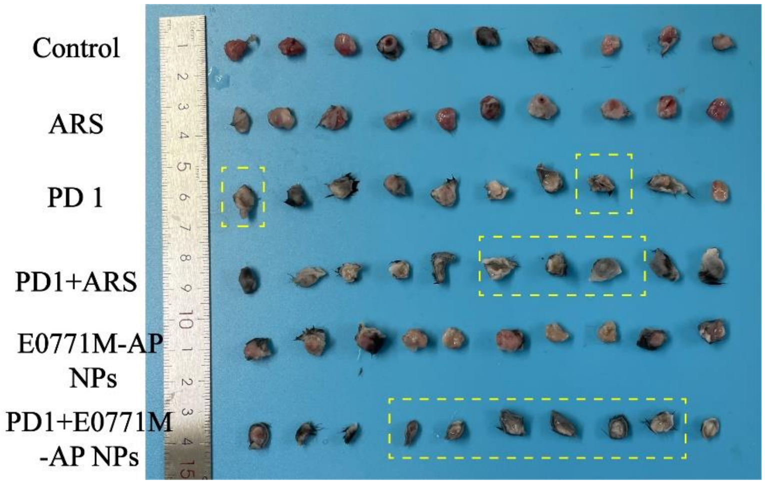

Fig.S23. Photograph of each tumor in different groups of mice on 18th day during the treatment period. Yellow dotted box: the eye (or hand) could not tell whether the breast pad contained tumor tissue.

24.

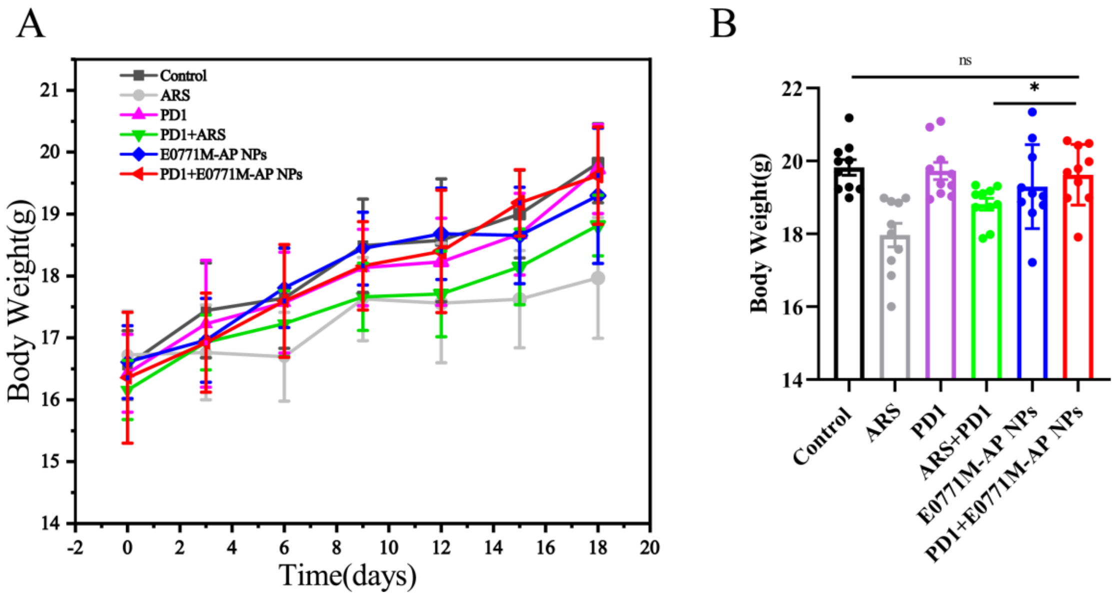

Fig.S24. (A) Body weight monitoring curves of different groups of mice during the 18-day treatment period (n=10). **(B)** Body weight of mice in different groups on the last day of treatment (n=10). Data expressed as mean ± SD.

25.

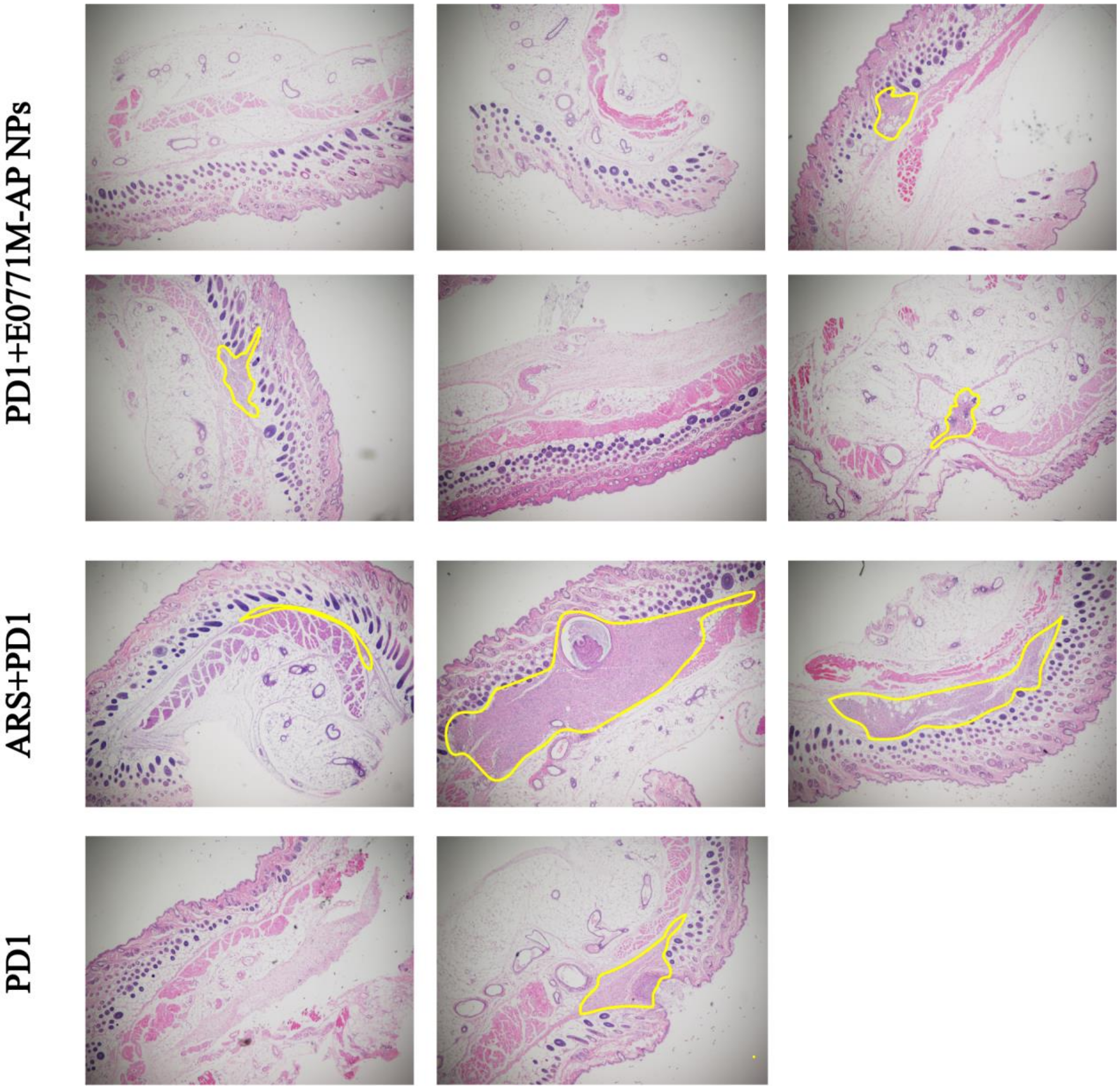

Fig.S25. H&E stained tissue of the breast pad that did not distinguish whether it contained tumor tissue. The yellow curve showed the margin of residual tumor tissue. (PD1+E0771M-AP NPs, n=6; ARS+PD1, n=3; PD1, n=2) (×40, magnification).

26.

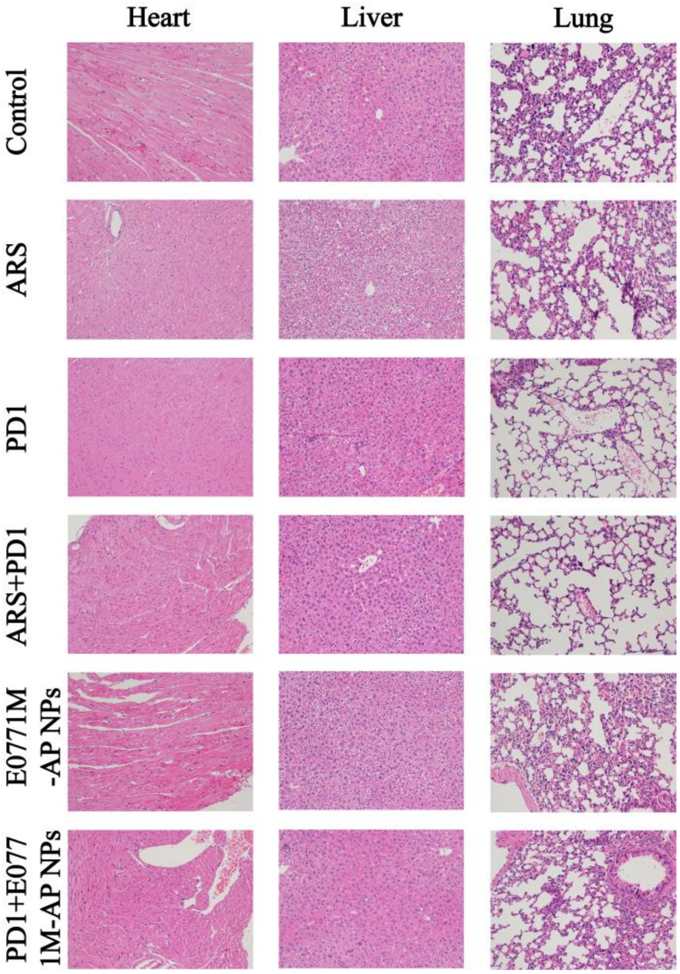

Fig.S26. Microscope images of H&E stained tissues in various organs (×400, magnification).

27.

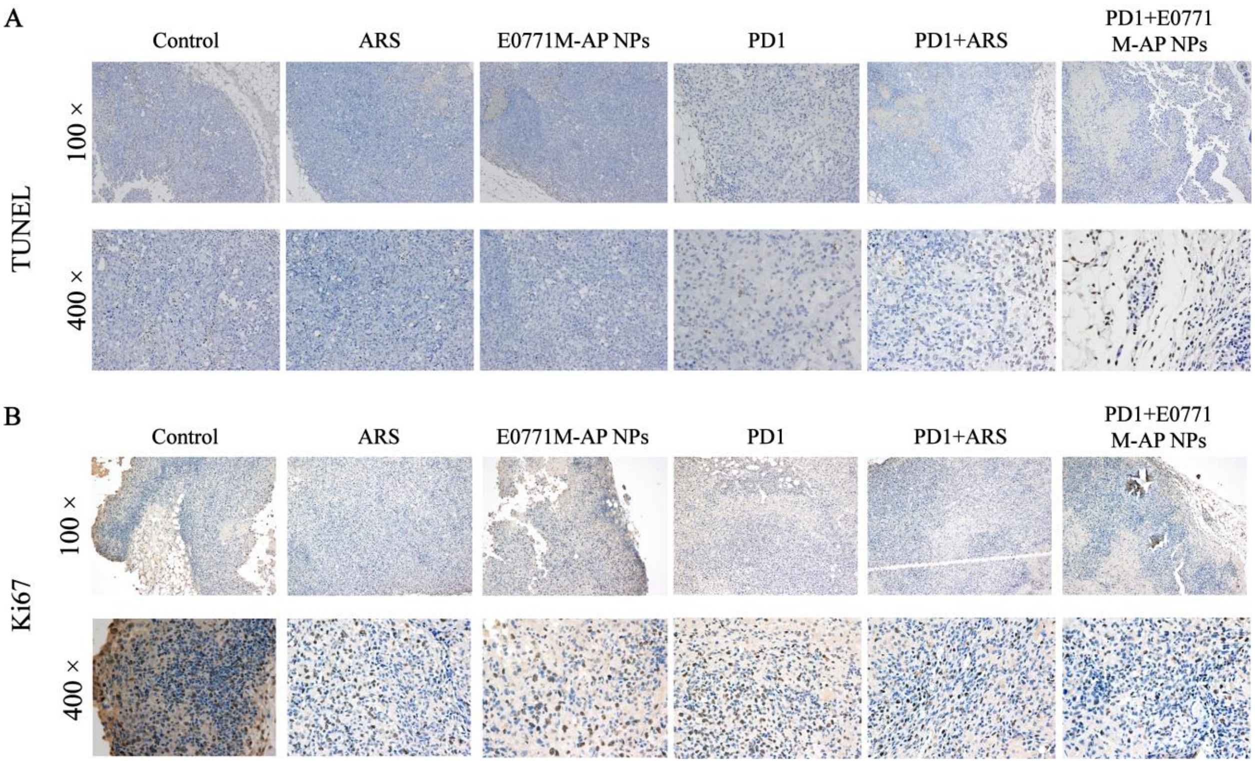

Fig.S27. (A) Representative microscope images of TUNEL stained tumor tissues in various groups (low magnification: 100×, high magnification: 400×); **(B)** Representative microscope images of Ki67 stained tumor tissues in various groups (low magnification: 100×, high magnification: 400×).

28.

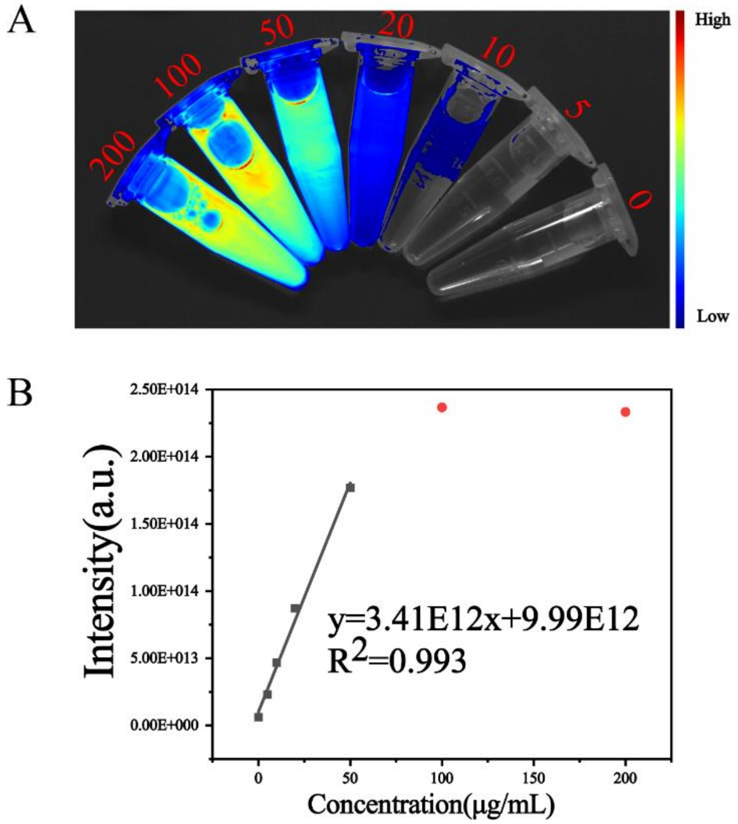

Fig.S28. (A) Fluorescence image of Cypate-loaded nanoparticles PBS solution with different concentrations (0, 5, 10, 20, 50, 100, 200μg/mL); **(B)** Concentration-Intensity standard curve of Cypate-loaded nanoparticles PBS solution at different concentrations. Red dots (where the concentration of nanoparticles is too high for Aggregation-Caused Quenching) were not selected for the fitting of the standard curve.

29.

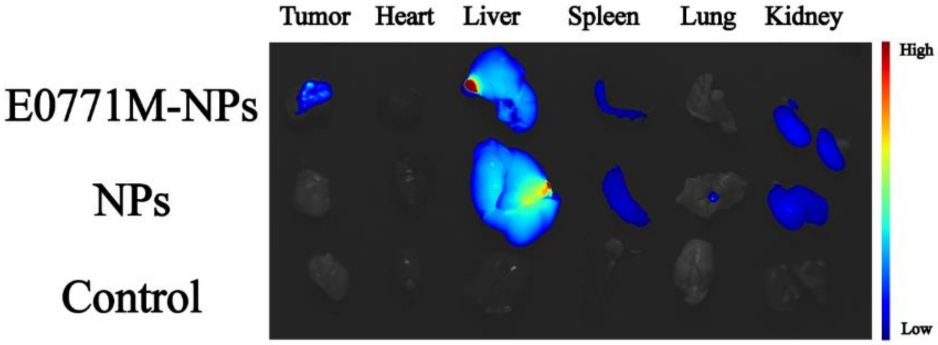

Fig.S29. Ex vitro fluorescence image of the major organs and tumors extracted from mice 6 hours after intravenous injection.

30.

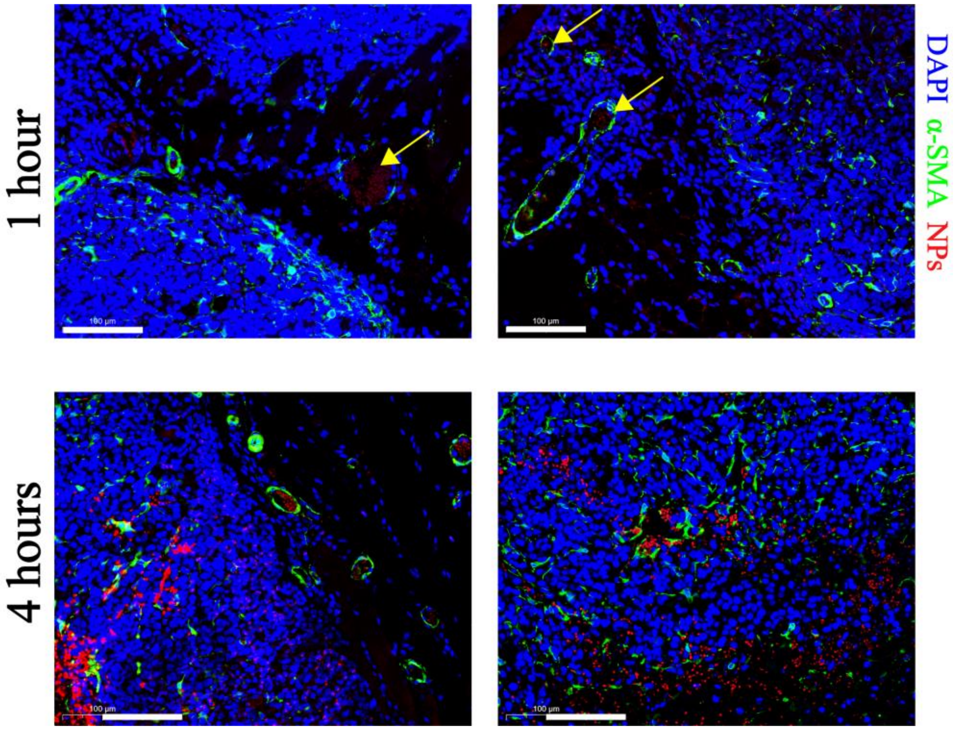

Fig. S30. Additional immunofluorescence images of NPs (red) accumulation in tumor tissue (blue) through tumor blood vessels (green). The yellow arrow indicated the position of blood vessels, scale bar: 100μm.

31.

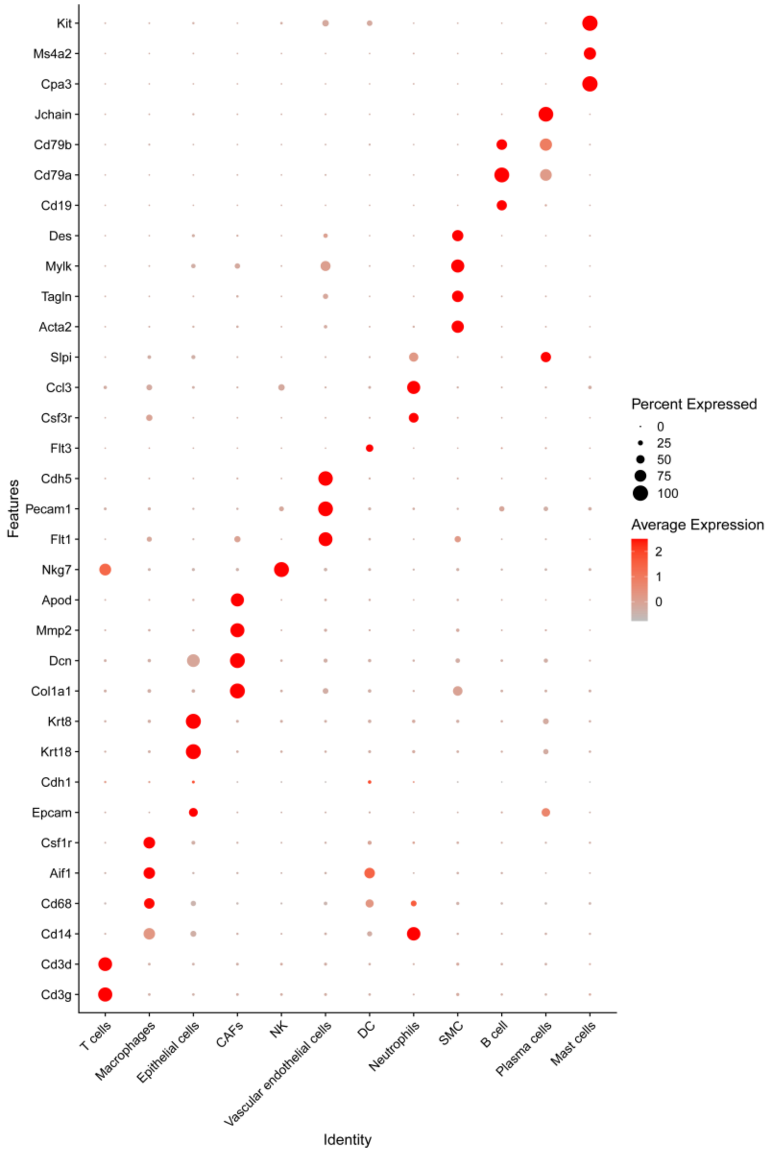

Fig. S31. Dot plots of all clusters differentially expressed gene.

32.

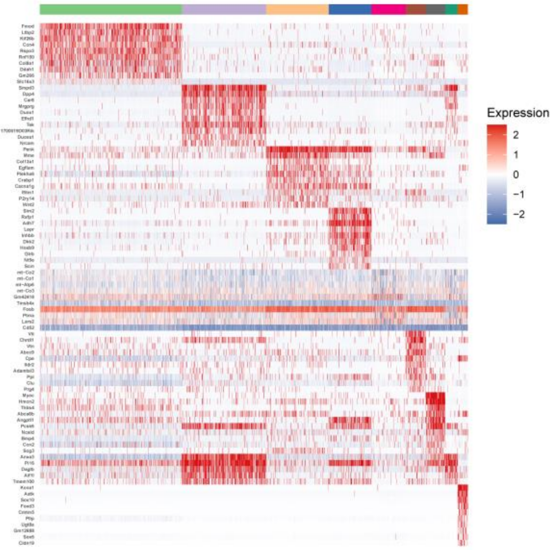

Fig. S32. Heatmap of top 10 differentially expressed genes in each CAFs cluster estimated by reproducibility-optimized test statistic.

33.

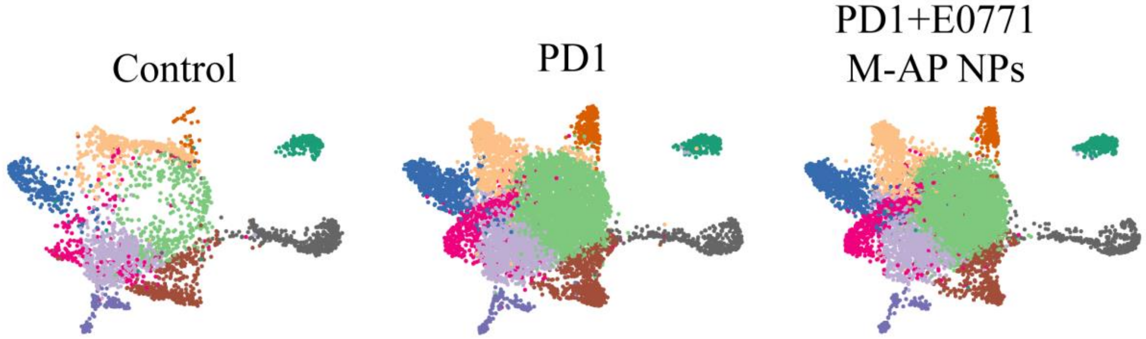

Fig. S33. An uniform manifold approximation and projection (UMAP) view of T cells, color-coded by assigned cell type.

34.

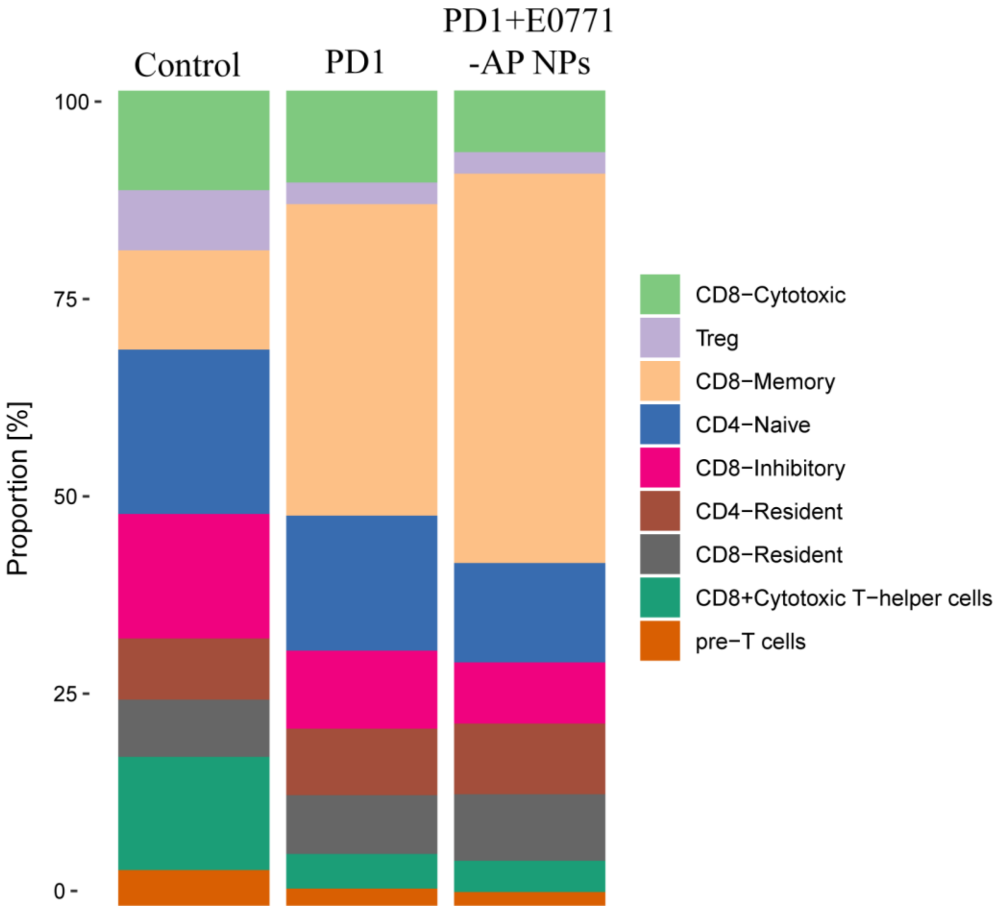

Fig. S34. Proportion of each T cell type in each group.

35.

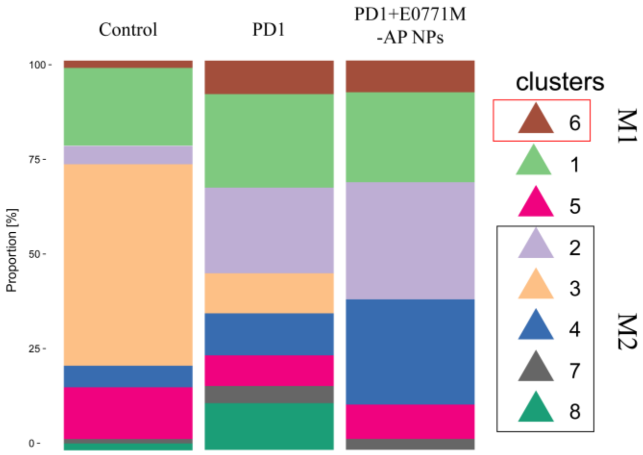

Fig. S35. Proportion of each macrophage cell type in each group.

36.

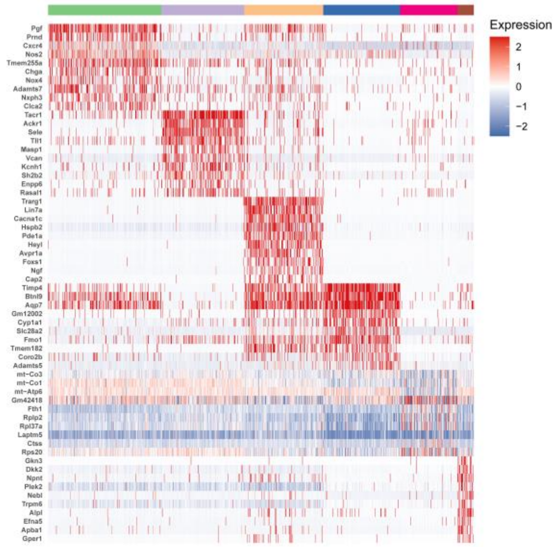

Fig. S36. Heatmap of top 10 differentially expressed genes in each vascular endothelial cell (vEC) cluster estimated by reproducibility-optimized test statistic.

Reference

[1] G. La Manno, R. Soldatov, A. Zeisel, E. Braun, H. Hochgerner, V. Petukhov, K. Lidschreiber, M.E. Kastriti, P. Lönnerberg, A. Furlan, J. Fan, L.E. Borm, Z. Liu, D. van Bruggen, J. Guo, X. He, R. Barker, E. Sundström, G. Castelo-Branco, P. Cramer, I. Adameyko, S. Linnarsson, P.V. Kharchenko, RNA velocity of single cells, Nature 560 (2018) 494–498.

[2] A.T. Lun, D.J. McCarthy, J.C. Marioni, A step-by-step workflow for low-level analysis of single-cell RNA-seq data with Bioconductor, F1000Research 5 (2016) 2122.
